# Supplementary material for: Versatile whole-organ/body staining and imaging based on electrolyte-gel properties of biological tissues
Source: Nat Commun. 2020 Apr 27;11:1982. doi: 10.1038/s41467-020-15906-5 (PMC7184626; doi:10.1038/s41467-020-15906-5)
Supplement: Supplementary file 1 — Supplementary Information [file 41467_2020_15906_MOESM1_ESM.pdf]

Supplementary information

Versatile whole-organ/body staining and imaging based on electrolyte-gel properties of biological tissues

Susaki et al.

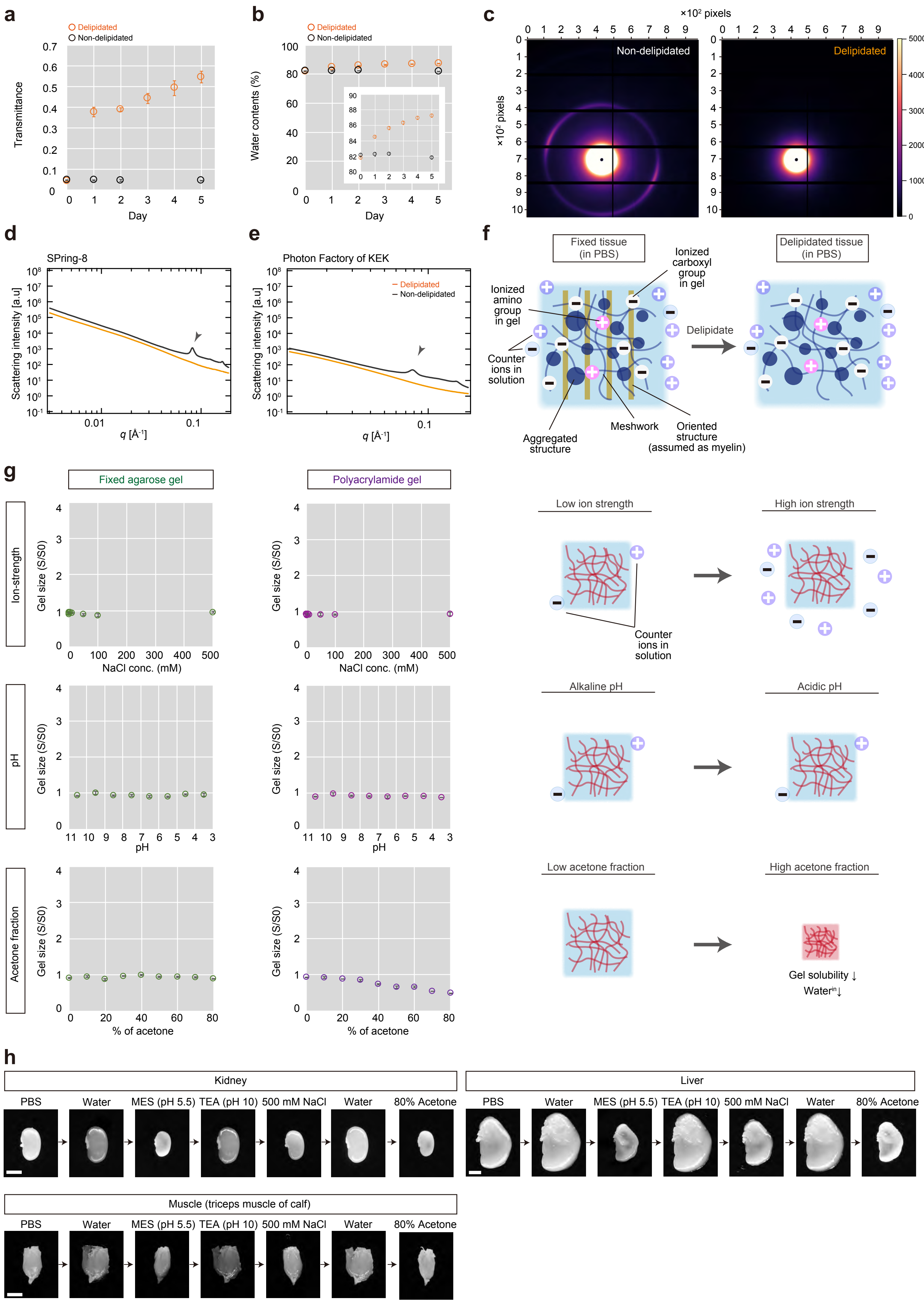

Supplementary Figure 1

Supplementary Figure 1. Fixed and delipidated tissue can be characterized as an electrolyte gel, related to Figure 1

a, **b** Changes in tissue transmittance and water content during delipidation, related to Fig. 1b and c. Inset: 80-90% of the y range.

c Two-dimensional intensity plot of scattered X-rays from the brain slices before and after delipidation, measured at SPring-8 (Harima, Japan). The unnormalized intensity (dynamic range of 0-1048576 stored as the 32-bit integer) is shown. Arrowheads indicate the portion of scattering related to an evenly spaced structure in the interior of the sample, assumed to correspond to the phospholipid layers of a myelin sheath. The sample was immersed in PB with 1 M NaCl. One pixel corresponds to an area of 172 × 172 μm^2. Black straight lines represent the gaps of the detector panels.

d One-dimensional profile of scattering intensity (I) as a function of the magnitude of the scattering vector (q) from the analysis of **c**. Arrowhead indicates the peak position corresponding to the assumed myelin structure, which disappeared after delipidation. The sample was immersed in PB with 1 M NaCl.

e The result in **d** was reproduced at a different facility, the Photon Factory of High Energy Accelerator Research Organization (KEK) (Tsukuba, Japan). The sample was immersed in PB with 1 M NaCl.

f Schematic of the fixed tissue before and after delipidation.

g Swelling-shrinkage curves of non-ionized artificial gels, including polyacrylamide gel used in tissue-hydrogel chemistry[1], related to Fig. 1g. The y-axis (S/S0) indicates the relative value of the gel area.

h Fixed and delipidated non-brain mouse tissues (a kidney, a lobe of the liver, and a triceps muscle of the calf) were treated with several chemical conditions as in Fig. 1a. MES: 2-(N-morpholino)ethanesulfonic acid, TEA, triethanolamine. Scale: 5 mm.

The values in **a**, **b** and **g** indicate the means ± SD (**a**: n = 4, **b:** n = 3, **g**: n = 3, biologically independent samples).

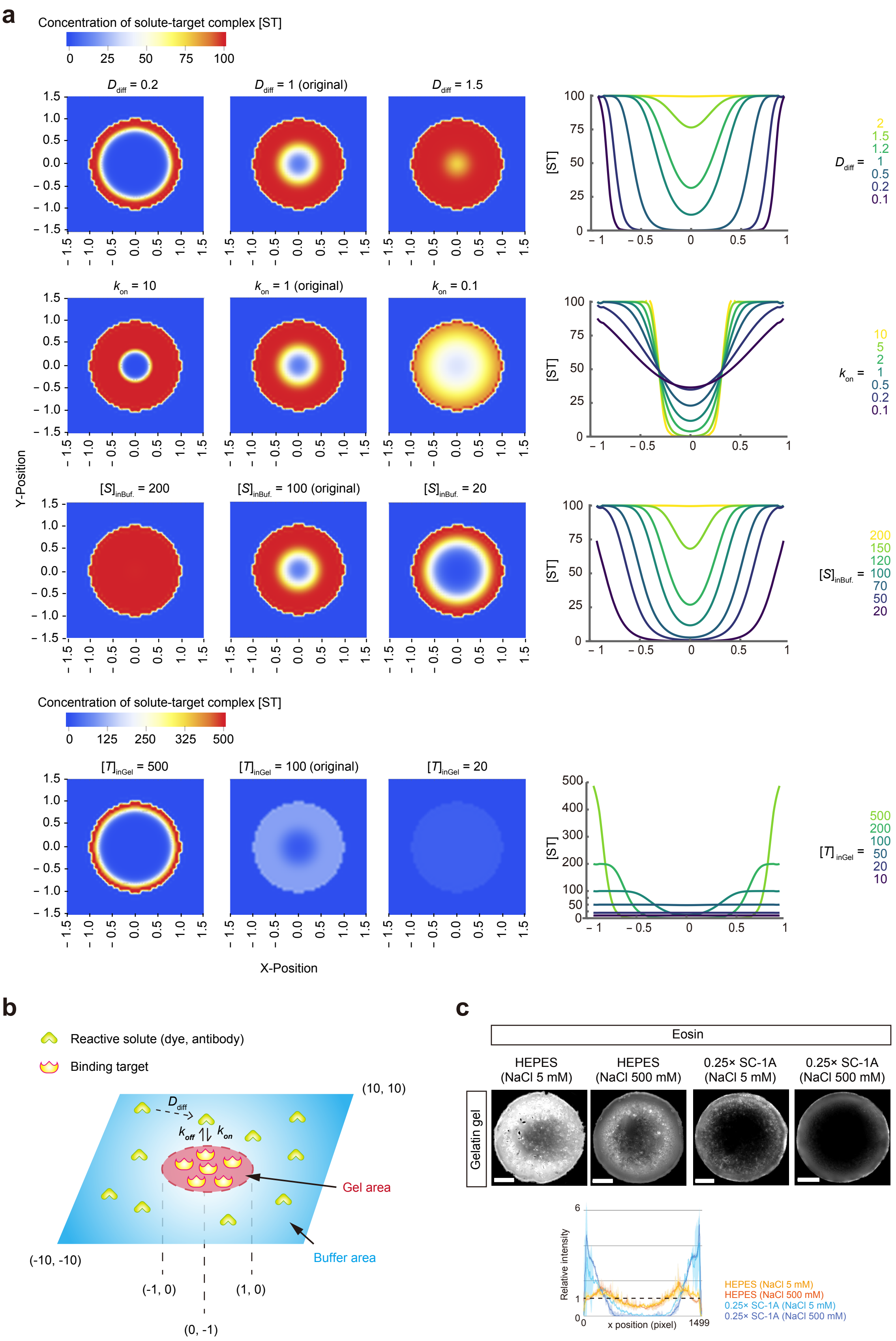

Supplementary Figure 2

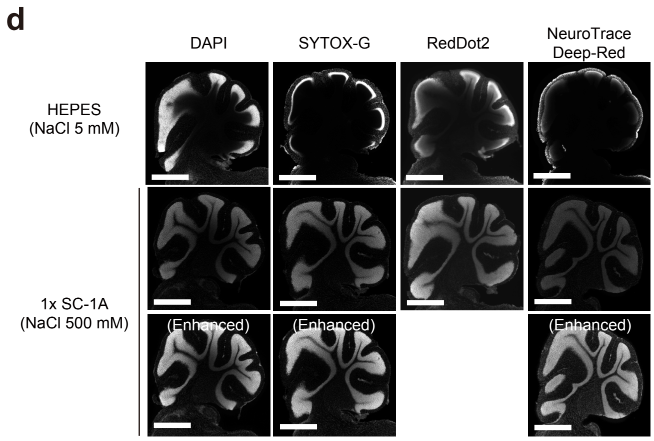

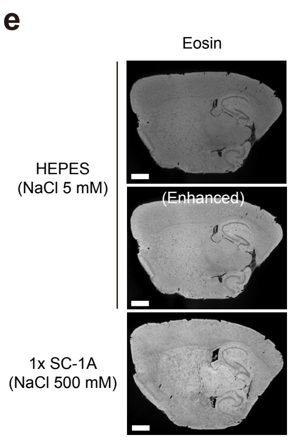

Supplementary Figure 2 (continued)

Supplementary Figure 2. Development of a versatile protocol for whole-organ/body dye staining, related to Figure 2

a Mathematical simulation of the diffusion-reaction model for evaluating the 3D staining pattern in a gel. When each parameter [diffusion constant (D_{diff}), binding constant (k_{on}), concentration of reactive solute in the buffer ([S]), concentration of interaction target in the gel ([T])] was altered from the original value, the interior staining pattern changed from a rimmed to a gradual pattern. All the simulations shown in this panel are the results of the simulation at time (t) = 0.5. Note that at t = 1, the solute-target complex ([ST]) is distributed almost homogeneously inside the circle for the system with the original parameter set.

b Schematic of the diffusion-reaction model in **a**.

c Representative results of gelatin gel staining with eosin, a negatively charged dye. Scale: 1 mm. The profile shows the mean intensity ± SD of the six diameter regions as in Fig. 2a.

d Comparison of 3D staining results in low-salt HEPES buffer and high-salt Sca*l*eCUBIC-1A (SC-1A). Fixed and delipidated tissues (cerebellar hemispheres) were 3D stained with various dyes. Then, they were sectioned to evaluate the infiltration of the dyes. Scale: 1 mm.

e A similar experiment in **d** using eosin. Scale: 1 mm.

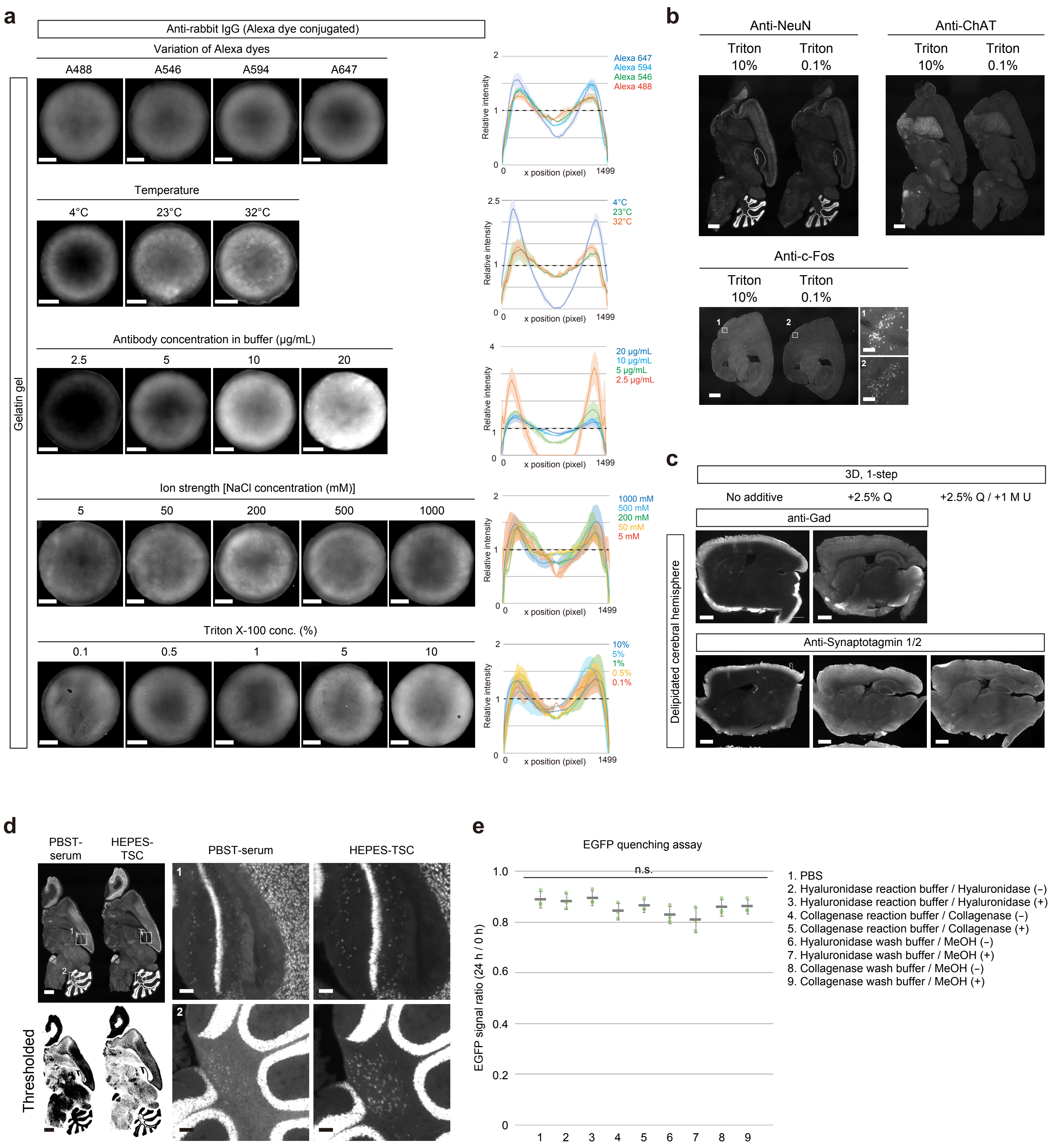

Supplementary Figure 3

Supplementary Figure 3. Development of a versatile protocol for whole-organ immunostaining, related to Figure 3

a Representative results of rabbit IgG-containing gelatin gel staining with anti-rabbit IgG. We used an A594-conjugated secondary IgG in all cases except for the “Variation of Alexa Fluor dyes” experiment. Scale: 1 mm. The profiles show the mean intensity ± SD of the six diameter regions as in Fig. 2a.

b Based on the results in **a**, we tested the effect of Triton X-100 concentration on tissue section staining. Delipidated brain sections were stained with the indicated antibodies at different Triton X-100 concentrations. Anti-NeuN (Millipore, MAB377), anti-ChAT (Millipore AB144) and anti-c-Fos (CST #2250 on MK-801-treated mouse brain) antibodies were tested. Scale: 1 mm (for entire sagittal sections) or 100 μm (for enlarged images).

c The use of the Sca*l*eCUBIC-1A (SC-1A) chemicals Quadrol (Q) and urea (U) improved antibody penetration in the tissue. One-step 3D staining with complexes of the indicated primary antibodies and secondary Fab (A594) was performed with or without additives. Then, antibody infiltration was evaluated by sectioning the tissue. Scale: 1 mm.

d Comparison of antibody staining buffers. HEPES-TSC buffer improved the signal-background ratio (SBR) over a conventionally used PBST buffer with serum (0.1% Triton X-100, 3% donkey serum). Anti-NeuN (Millipore, MAB377) antibody was tested. Scale: 1 mm (for entire sagittal sections) or 100 μm (for enlarged images).

e EGFP quenching assay in the limited enzyme reaction and washing buffers. Statistical significance was evaluated by one-way ANOVA ($F_{8,18}$ = 2.1648, P = 0.08279). The values indicate the mean ± SD (n = 3, independent experiments) of the fluorescent signal ratio (24 h/0 h, dot). n.s., not significant.

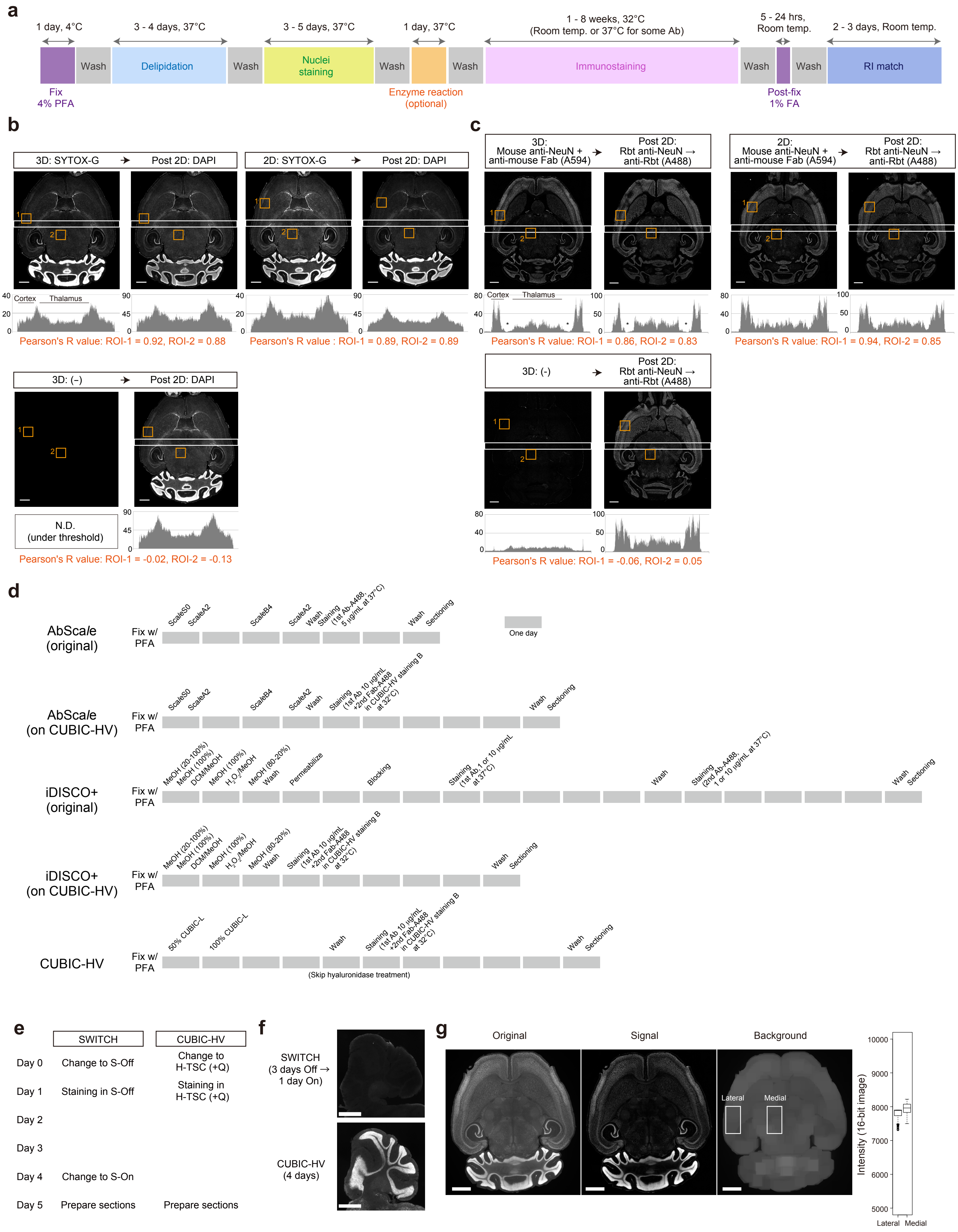

Supplementary Figure 4

Supplementary Figure 4. Validation of the CUBIC-HV protocol for whole-organ immunostaining, related to Figure 3

a Overview of the clearing, staining and RI matching of CUBIC-HV1.0, including the optional enzyme reaction step.

b Evaluation of whole adult mouse brain 3D staining with SYTOX-G. After 3D staining, the sample was sectioned and restained with DAPI (post-2D). As the control, a nonstained section was stained with SYTOX-G followed by DAPI (2D and post-2D). Intensity profiles in the middle part of the section (white rectangles) and Pearson's R values (no threshold) of the colocalization at the exterior (ROI-1) and interior (ROI-2) regions (orange squares) are shown for comparison. Scale: 1 mm.

c A similar evaluation of whole adult mouse brain 3D staining with a complex of mouse anti-NeuN antibody and secondary Fab (A594) fragment. After 3D staining, 2D sections were prepared and restained with rabbit anti-NeuN antibody followed by anti-rabbit secondary antibody (A488) (post-2D)[2]. As the control, a nonstained section was used and underwent the same staining procedures (2D and post-2D). The intensity profiles and Pearson's R values are shown as in **b**. Asterisks indicate regions of enlarged ventricles specifically observed in the sample. Scale: 1 mm.

d Procedures of the 3D staining tested in Fig. 3h.

e, **f** Comparison of anti-NeuN 3D staining with SWITCH-mediated antibody labeling (Figure 6C in Murray et al. 2015[3]) and CUBIC-HV over the same timescale. S-Off and S-On indicate SWITCH-Off and -On buffers (0.5 mM SDS in PBS or PBST, respectively). H-TSC (+Q) indicates HEPES-TSC with Quadrol. Scale: 1 mm.

g Assessment of light-sheet absorption in the tissue. The same imaging data were used in Fig. 3i and j. The box plots (minimum, 25th percentile, median, 75th percentile and maximum) of signal intensities at the indicated position (200 × 350 pixels) are shown. Scale: 2 mm.

a

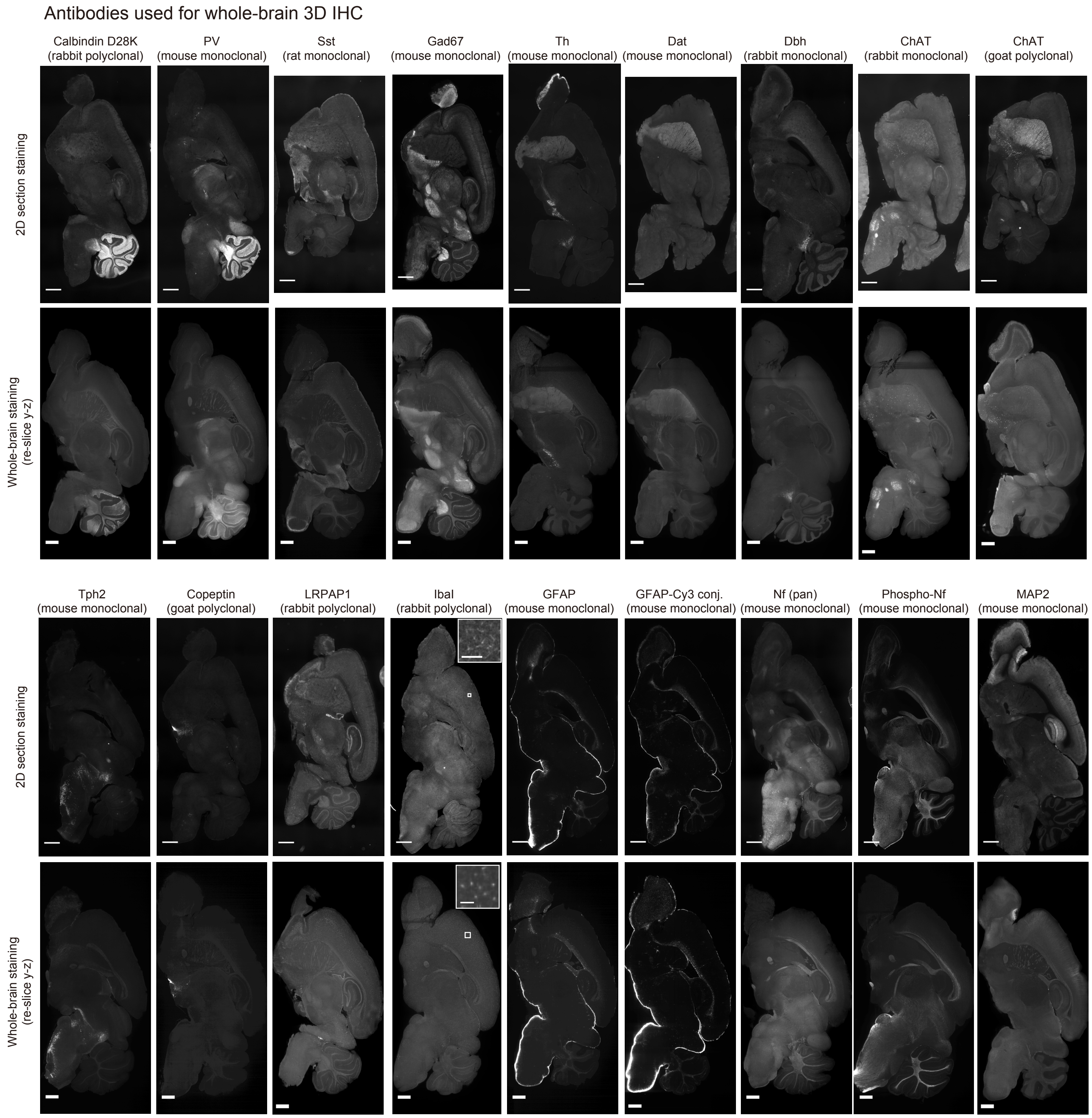

Supplementary Figure 5

a (continued)

Antibodies used for whole-brain 3D IHC (continued)

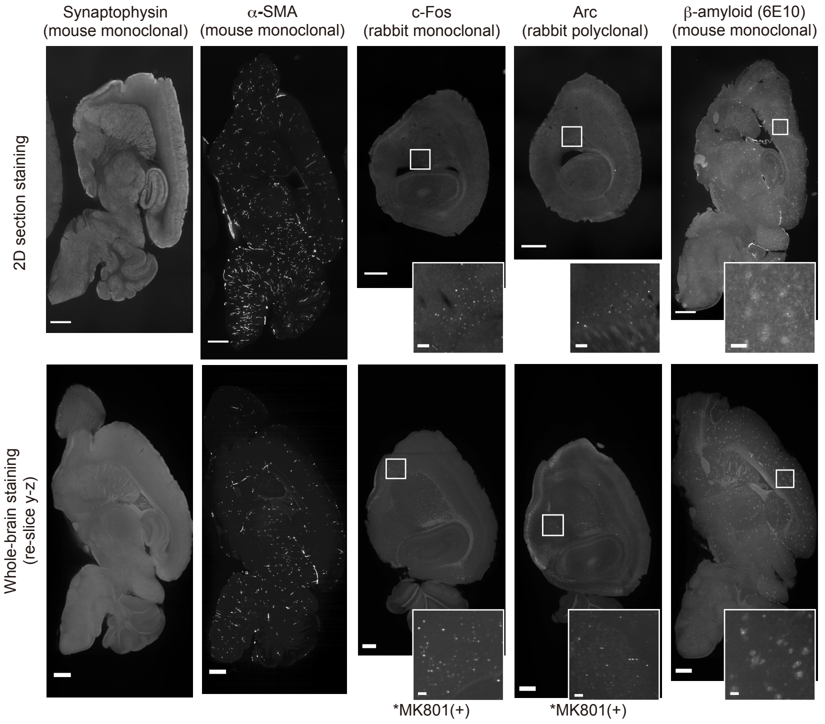

b

Other CUBIC-compatible antibodies (untested or unsuitable for whole-brain staining)

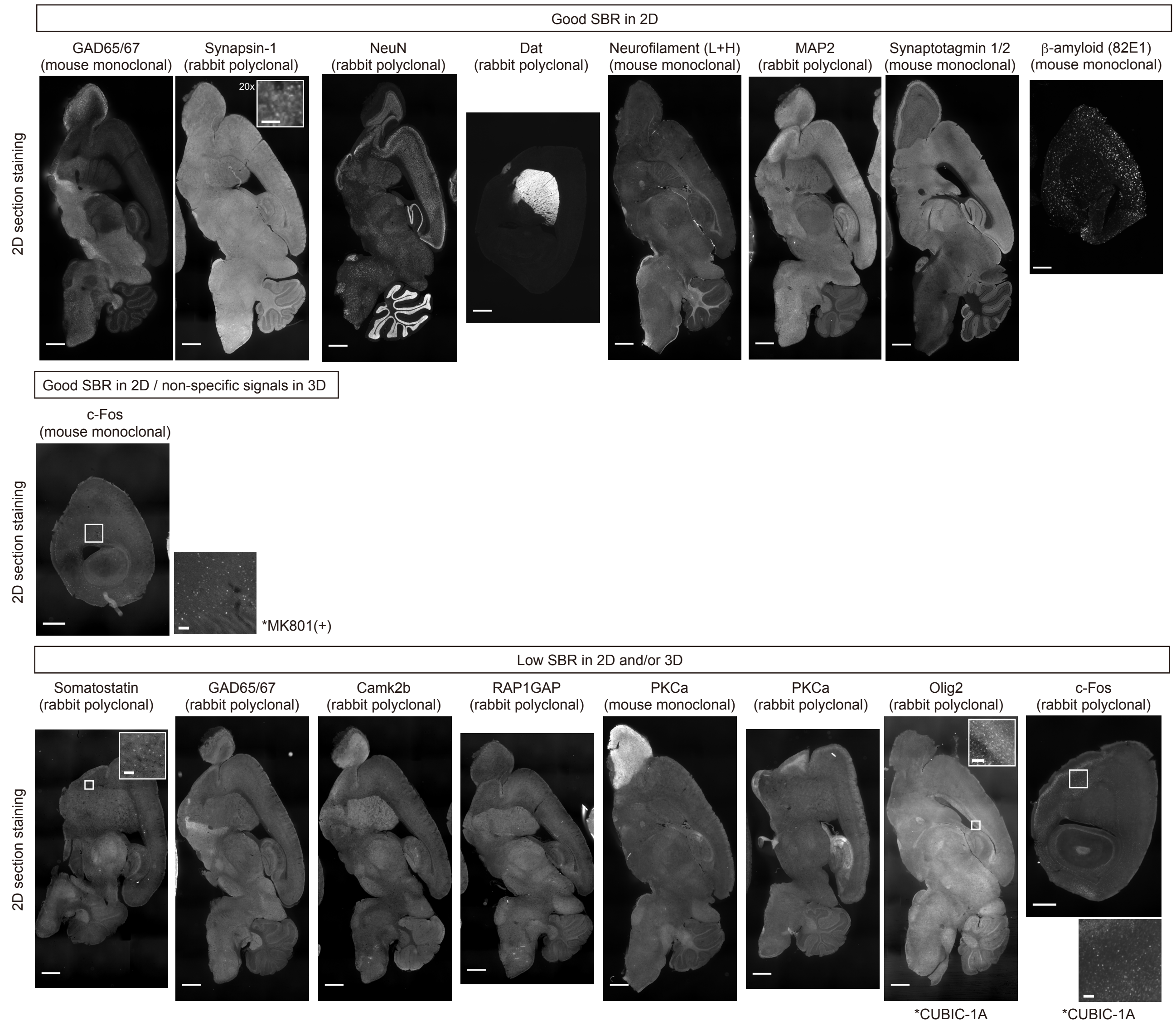

Supplementary Figure 5 (continued)

Supplementary Figure 5. Immunostaining results tested for CUBIC-HV, related to Figure 4

a Reconstituted sagittal sections of 3D immunostaining data and corresponding 2D immunostaining results, related to the data in Figs. 4-9 and Supplementary Data 1. The images were prepared with Fiji/ImageJ. Note that the current protocol occasionally caused nonspecific signals in white matter regions, which were attributable to the postfixation step. We will solve this issue in a future update. Scale: 1 mm (whole sagittal images), 0.1 mm (insets).

b 2D immunostaining results for antibodies excluded from 3D staining processes. Scale: 1 mm (whole sagittal images), 10 μm (inset of Synapsin-1) or 0.1 mm (other insets).

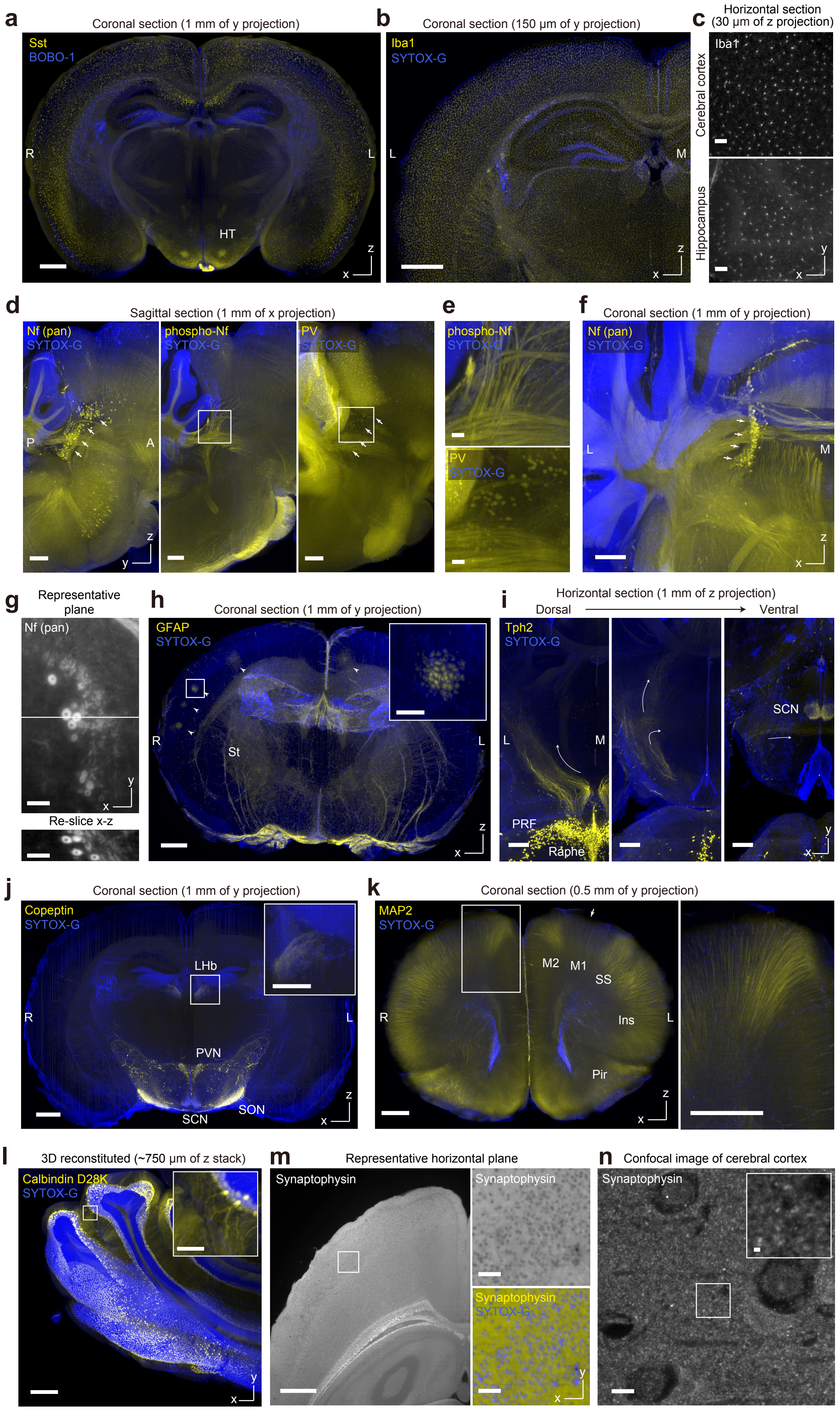

Supplementary Figure 6

Supplementary Figure 6. CUBIC-HV is applicable to various antibodies and dyes, related to Figure 4

a, b Reconstituted sections of BOBO-1/Sst-labeled (**a**) or SYTOX-G/Iba1-labeled (**b**) whole brains. HT: hypothalamus, R in **a**: right, L in **a**: left. L in **b**: lateral, M in **b**: medial. Scale: 1 mm.

c Magnified images of the brain in **b** at the indicated regions. Scale: 0.1 mm.

d Reconstituted sections of SYTOX-G/Nf (pan)-, SYTOX-G/phospho-Nf or SYTOX-G/PV-labeled whole brains. Arrows: Nf (pan)- and PV-positive but phospho-Nf-negative large globular cells in the mesencephalic trigeminal nucleus. P: posterior, A: anterior. Scale: 0.5 mm.

e Enlarged images at the position indicated in **d**. Scale: 0.1 mm.

f Another reconstituted section of the data in **d**. L: lateral, M: medial. Scale: 0.5 mm.

g Magnified and resliced images of globular cells. White line: resliced position. Scale: 0.1 mm.

h A reconstituted section of the SYTOX-G/GFAP-labeled whole brain. Arrowheads: GFAP-positive cell foci in the cerebral cortex (enlarged in inset). St: striatum, R: right, L: left. Scale: 1 mm and 0.2 mm (inset).

i Reconstituted serial sections of the SYTOX-G/Tph2-labeled whole brain. Arrows: afferent projections from the serotonergic nuclei in the pons. PRF: pontine reticular formation, SCN: suprachiasmatic nucleus, L: lateral, M: medial. Scale: 1 mm.

j, k Reconstituted sections of the SYTOX-G/copeptin-labeled (**j**) and SYTOX-G/MAP2-labeled (**k**) whole brains. White box (**j**): lateral habenula (LHb), showing afferent projections of the AVP neuron (inset). Arrow (**k**, left panel): region with relatively sparse MAP2-positive dendrites. White box (**k**, left panel): enlarged region in the right panel. PVN: paraventricular hypothalamic nucleus, SON: supraoptic nucleus, SCN: suprachiasmatic nucleus, M1: primary motor cortex, M2: secondary motor cortex, SS: somatosensory cortex, Ins: insular cortex, Pir: piriform cortex, R: right, L: left. Scale: 1 mm and 0.5 mm (inset in **j**).

l A magnified and 3D reconstituted cerebellar image of the SYTOX-G/calbindin D28K-labeled whole brain. White box: position of the enlarged image (inset). Scale: 0.5 mm or 0.1 mm (inset).

m A magnified cortical image of the SYTOX-G/synaptophysin-labeled whole brain. White box: region enlarged in the right panels, showing the immunostaining signals outside the nuclei. Scale: 1 mm (left panel) or 0.1 mm (right panels).

n Another CUBIC-L-treated brain was 3D stained with anti-synaptophysin antibody and imaged with a confocal microscope equipped with a 60×/1.35 objective lens and 2×

digital zoom. The magnified image demonstrated the synaptic staining pattern. White box: position of the enlarged image (inset). Scale: 10 μm or 1 μm (inset).
The images were recaptured from the data in Fig. 4a (**a**, **b**, **d**-**f**, **h**-**k**) or acquired by another imaging modality (**c**, **g**, **l**, **m**). They were processed with Imaris software (**a**, **b**, **d**, **h**-**l**) or Fiji/ImageJ (**c**, **g**, **m**). The voxel sizes are 8.3 × 8.3 × 9 μm^3 (**a**, **b**, **d**, **h**-**k**) or 2.5 × 2.5 × 3 μm^3 (**c**, **g**, **l**, **m**).

a

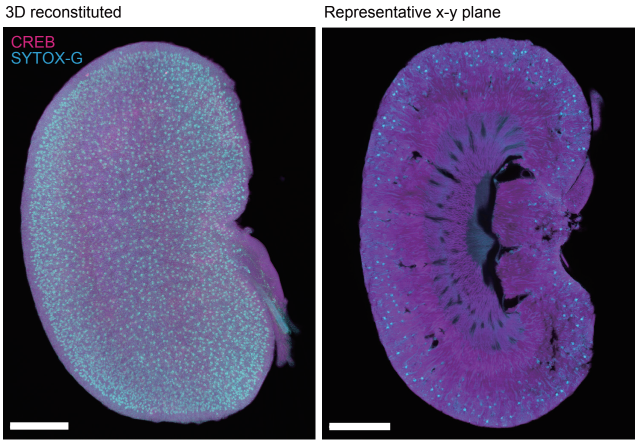

Supplementary Figure 7

Supplementary Figure 7. CUBIC-HV is applicable to organs other than the brain, related to Figure 4

a Results of whole-kidney staining and LSFM imaging with anti-CREB antibody (magenta) and SYTOX-G (cyan). The data were reconstituted with Imaris software. The voxel size is 6.5 × 6.5 × 7 μm^3. Scale: 2 mm.

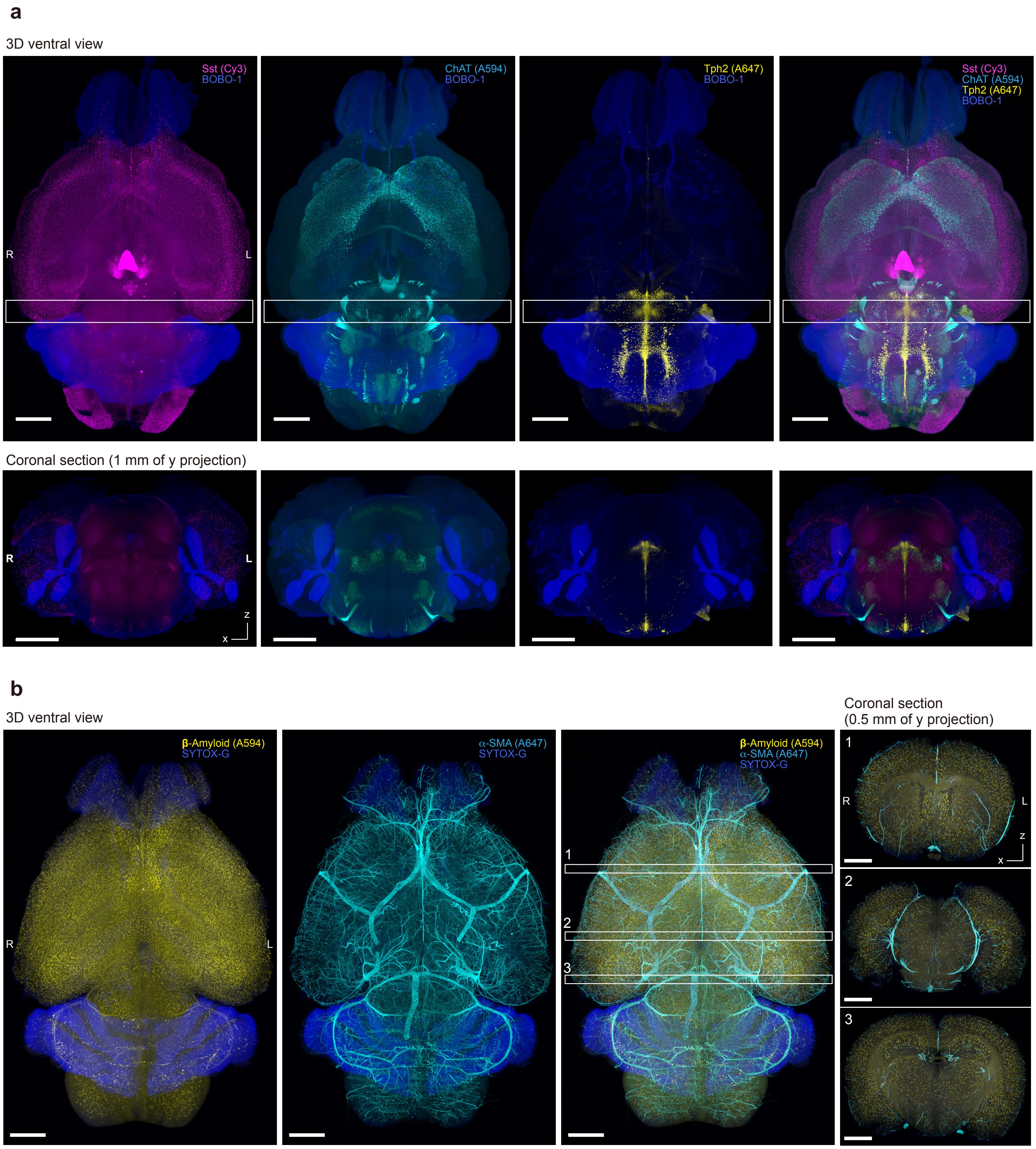

Supplementary Figure 8

Supplementary Figure 8. CUBIC-HV enables multicolor whole-organ staining and imaging, related to Figure 5

a Multichannel whole-brain imaging of BOBO-1, Sst (Cy3), ChAT (A594) and Tph2 (A647) costained samples. Reconstituted coronal (x-z) sections at the indicated position are also shown. Voxel size: 8.3 × 8.3 × 9 μm^3. Scale: 2 mm.

b Multichannel whole-brain imaging of the SYTOX-G, β-amyloid (A594) and α-SMA (A647) costaining samples. A 9-month-old mouse model of Alzheimer's disease ($\textit{App}^{NL\text{-}G\text{-}F}$ knock-in mouse[4]) was used. The reconstituted coronal (x-z) sections at the indicated positions are also shown. Voxel size: 8.3 × 8.3 × 9 μm^3. Scale: 2 mm.

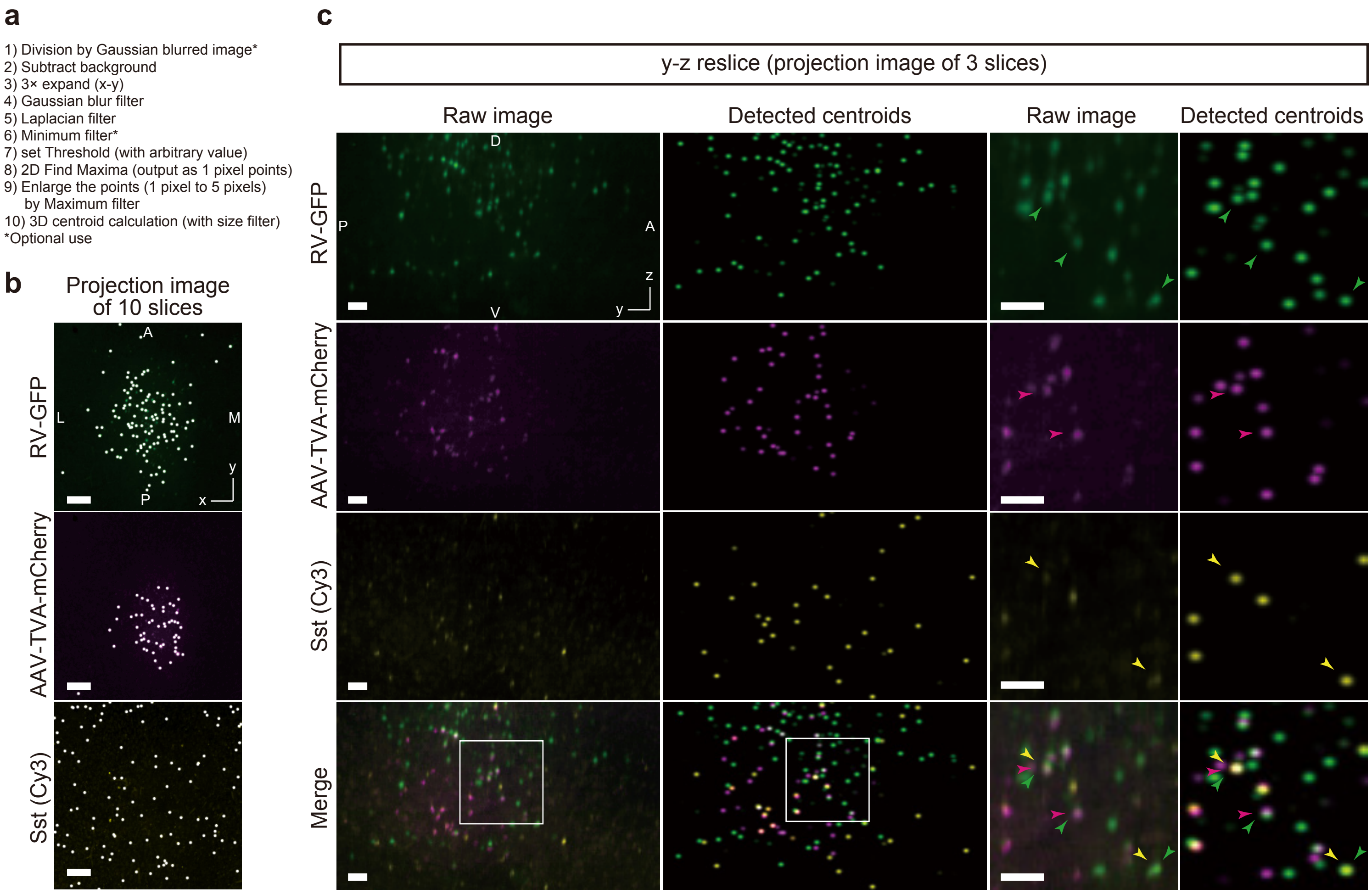

Supplementary Figure 9

Supplementary Figure 9. CUBIC-HV allows whole-organ cellular circuit analysis, related to Figure 7

a Overview of the cell detection algorithm for RV data.

b Calculation results for the injected region (x = 250 pixels, y = 250 pixels, z = 10 slices) from the RV- and Sst-labeled brain in Fig. 7b-j. The detected centroids (white) were merged with the corresponding labeled cells. Note that the centroids of cells located in the top and bottom of the stack were calculated outside the range. The sensitivity and positive predictive value (PPV), respectively, of the algorithm are as follows: 114/128 = 89.1% and 114/115 = 99.1% for GFP+ cells; 54/73 = 74.0% and 54/55 = 98.2% for mCherry+ cells; and 110/169 = 65.1% and 110/115 = 95.7% for Sst+ cells. A: anterior, P: posterior, L: lateral, M: medial. Scale: 0.2 mm.

c Reconstituted y-z images of the raw data and the calculated centroids. The raw data images, corresponding to the data from Fig. 7g-j, were differently recropped with Fiji/ImageJ. The dual- and triple-labeled cells were reconstituted with the calculated centroids. A: anterior, P: posterior, D: dorsal, V: ventral. Scale: 0.1 mm.

a

1) Subtract background
2) 3× expand (x-y-z)
3) 3D Minimum filter
4) 3D Gaussian blur filter
5) set Threshold
 (with arbitrary value)
6) 3D Find Maxima

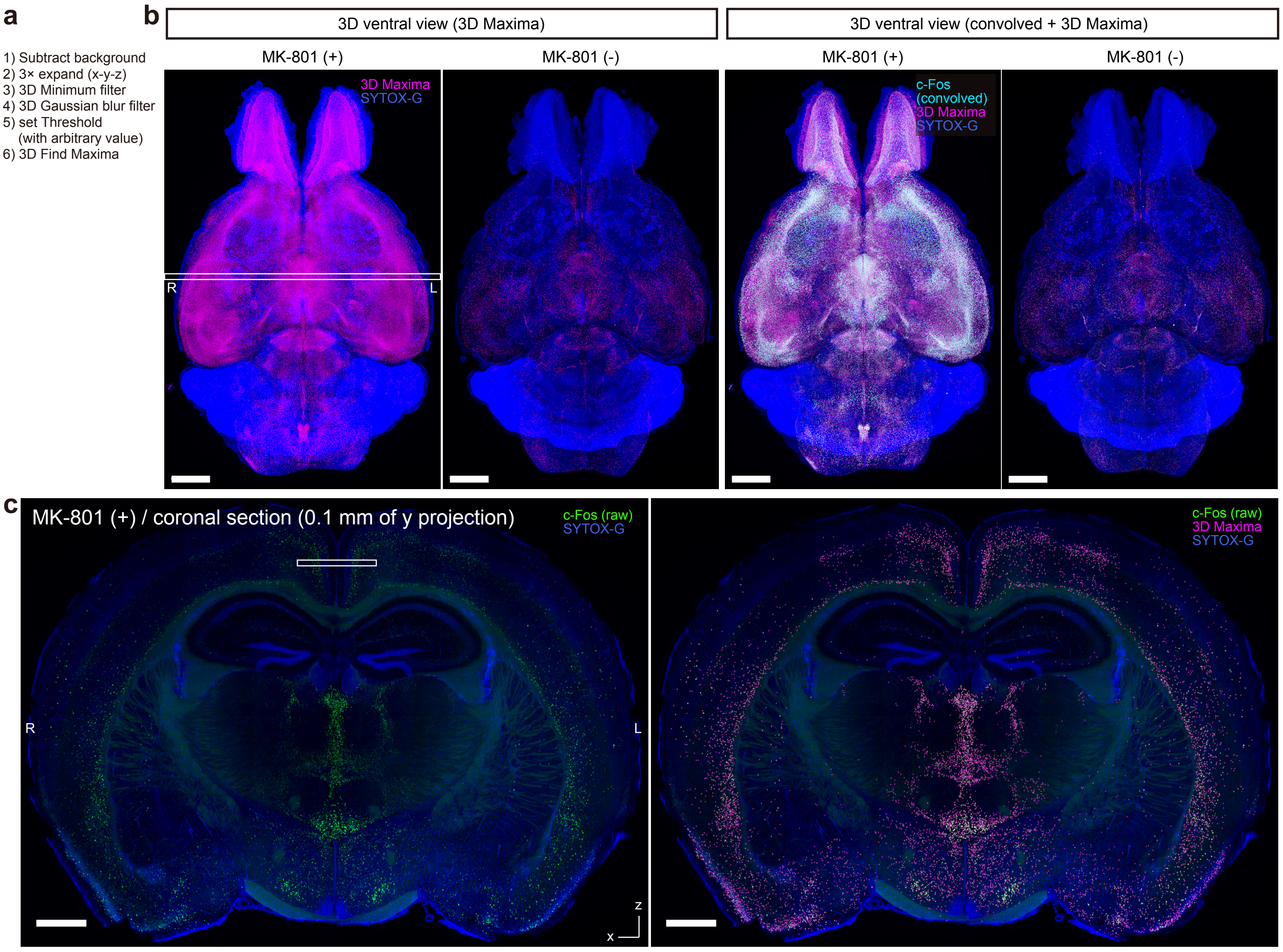

Supplementary Figure 10

Supplementary Figure 10. CUBIC-HV allows whole-organ cellular function analysis, related to Figure 8

a Overview of the cell detection algorithm for c-Fos immunolabeling data.

b Calculation results for the whole-brain c-Fos-labeled data in Fig. 8b. The detected 3D Maxima were merged with the corresponding c-Fos signals (enhanced by a convolutional filter as in Fig. 8c). The white box in the left panel indicates the position of the reconstituted coronal sections in **c**. R: right, L: left. Scale: 2 mm.

c The reconstituted coronal sections at the position indicated in **b**. The white box indicates the area used to evaluate the sensitivity (382/491 = 77.8%) and PPV (381/381 = 100%) of the algorithm. R: right, L: left. Scale: 1 mm.

Note that while these algorithms result in a high PPV, they are relatively insensitive because the cutoff threshold applied to remove background signals also eliminated weak, positive signals. This issue will be improved in future studies by improving the SBR from the 3D immunostaining protocol and implementing a machine learning-based algorithm.

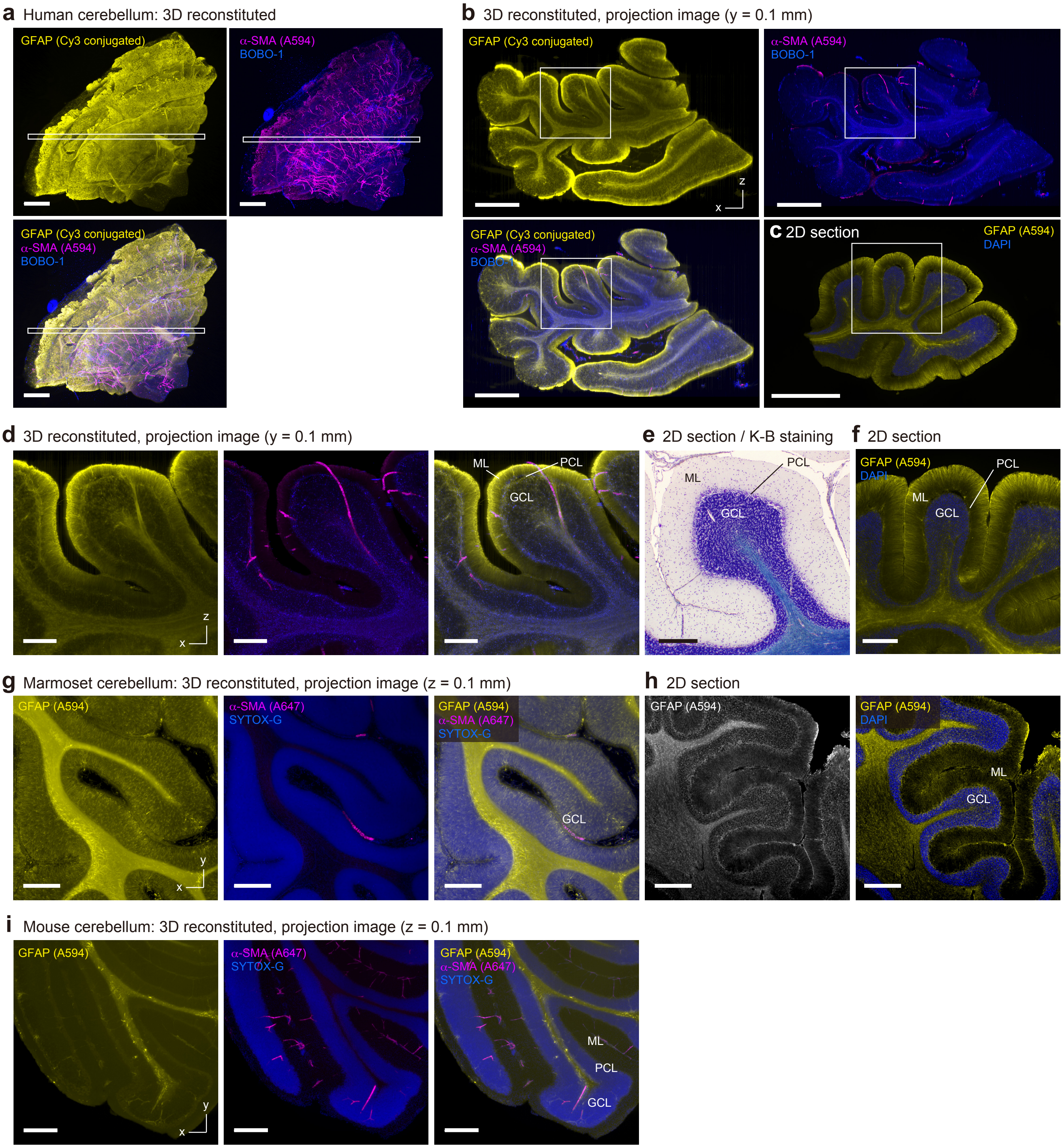

Supplementary Figure 11

Supplementary Figure 11. CUBIC-HV enables organ-level comparison of rodent and primate brains, related to Figure 9

a The 3D staining results of an ~1 cm^3 block of human postmortem cerebellum. Note that we used another GFAP antibody (Cy3-conjugated anti-GFAP from Sigma-Aldrich), expecting an improved penetration compared to a complex of primary IgG and secondary Fab. White boxes indicate the region shown in **b**. Scale: 2 mm.

b The reconstituted x-z section at the position indicated in **a**. White boxes indicate the region magnified in **d**. Scale: 2 mm.

c The remaining cerebellar part of the same human specimen was delipidated and sectioned. The physical sections were 2D stained with anti-GFAP-Cy3 antibody and DAPI. The GFAP staining pattern was comparable with the 3D staining data in **b**. The white box indicates the region enlarged in **f**. Scale: 2 mm.

d Magnified images at the indicated region in **b**. Scale: 0.5 mm.

e The Klüver-Barrera (K-B) staining result of the remaining cerebellar part of the same human specimen, showing the layer structure for reference. Scale: 0.5 mm.

f The enlarged image at the region indicated in **c**. Scale: 0.5 mm.

g Magnified cerebellar images of the marmoset sample in Fig. 9. Scale: 0.5 mm.

h Another marmoset cerebellum specimen was delipidated and sectioned. The physical sections were 2D stained with anti-GFAP antibody and DAPI. The GFAP signal was obvious in the granular layer and the white matter as in **g**. Scale: 0.5 mm.

i Magnified cerebellar images of the mouse sample in Fig. 9. Scale: 0.5 mm.

Note that different orientations of the sections [x-z in the human case in **b**, x-y in the marmoset and mouse cases in **g** and **i**] could be compared because of the almost isotropic voxel resolution of the z-stack images. ML, molecular layer; PCL, Purkinje cell layer; GCL, granule cell layer. Voxel sizes are 8.3 × 8.3 × 9 μm^3 (**a**) or 2.5 × 2.5 × 3 μm^3 (**d**, **g**, **i**).

Supplementary Discussion

Common and distinct physicochemical properties of biological tissues and artificial gels

The presence of ionic interactions in the tissue gels was suggested by the results of swelling-shrinkage profiles. First, in the NaCl solution and acetone fraction, the tissue gels showed more substantial dynamics of volume change due to ionic interactions (Fig. 1g) than the non-ionic fixed agarose or polyacrylamide gels (Supplementary Fig. 1g). Second, both the tissue and the fixed gelatin gel showed a volume phase transition during pH change. This behavior could be attributed to the properties of the amphoteric ion gel[5, 6]. However, the tissue markedly shrank more at acidic pH levels than the gelatin gel, suggesting differences in the electrostatic interactions, hydrophobic interactions, or structural strength of the polymers within the gels. In that sense, the tissue might be more similar to artificial copolymer gels such as NIPA/AAc gel or MAPTAC/AAc gel[5], both of which are categorized as amphoteric ion gels with internal hydrophobic interactions.

The *I*-*q* profile from the SAXS analysis provides other aspects of the tissue as an electrolyte gel (Fig. 1e, f). The power-law distribution ($D \approx 2$) of the *I*-*q* profile reflects the fractal nature of the sample having a highly heterogeneous hierarchic structure[7, 8]. The broad peak in the profile suggests that long-range repulsive interactions between the polymers are formed, which are caused by the electrostatic interactions between the polypeptides and may be helped by their flexible nature. In addition, the range of the peak (corresponding to the size 15 - 30 nm) could be considered the practical mesh size of the sample.

Essential 3D staining conditions found in the gelatin gel staining assays

Modulation of the interactions between the reactive solute and the gel had a marked effect on the degree of 3D staining. Given that the nuclear stains used in this study have a cationic charge and interact with negatively charged polymers (in this case, both DNA and the fixed polypeptide gels), high concentrations of salt could partially neutralize the ionic interactions and promote penetration of the stains (Fig. 2b, d). Conversely, penetration of the negatively charged dye eosin was faster and salt-independent due to the absence of such ionic interactions (Supplementary Fig. 2c, e). We also found that the use of chemicals in Sca*l*eCUBIC-1A (a cocktail of Quadrol, Triton X-100 and urea) was required for 3D staining of the cell-permeable lipophilic stain SYTO 16 and some antibodies (Fig. 2c, e; Supplementary Fig. 3c). Triton X-100 and Quadrol may prevent

some interactions, including hydrophobic and nitrogen-mediated coordination bonds[9-11]. Urea has a well-known ability to disrupt hydrogen bonds and is already used in the AbSca*l*e protocol[2]. Further chemical screening and profiling in the future will contribute to the discovery of more suitable additives and underlying chemical principles for efficient penetration of dyes and antibodies.

The two-step staining procedure with primary and then secondary antibodies seems better to be avoided in three-dimensional immunostaining. After using a primary antibody, the gel became saturated with a high concentration of antigen for the secondary antibody. Binding of the secondary antibody to the highly abundant primary antibody might block the penetration of the secondary antibody (Fig. 3a, c, d). This explanation can account for why AbSca*l*e, which recommends the use of dye-conjugated primary antibodies, results in better NeuN staining than iDISCO+ in our experience (Fig. 3h; Supplementary Fig. 4d). The current CUBIC-HV protocol applies either a dye-conjugated primary antibody or a complex of primary antibody and secondary Fab fragment to get around the two-step staining procedure. We propose the latter method for applying the protocol to a broader range of primary antibodies.

The idea of temporarily controlling interactions (k_{on} and k_{off} of the binding term) during 3D staining has been proposed and tested in SWITCH-mediated antibody labeling[3]. To temporarily control antibody binding, the SWITCH protocol was implemented with a two-step staining procedure involving the addition and removal of SDS (SWITCH-Off and -On, Figure 6C in the reference). In principle, the idea seems to increase dye and antibody penetration in tissue samples. However, when we compared this two-step implementation with our one-step implementation, the NeuN signal was not observed in the SWITCH-stained cerebellum, while we obtained the expected 3D staining result with CUBIC-HV over the same staining period (Supplementary Fig. 4e, f). Therefore, potential concerns might exist in their original two-step implementation, such as insufficient recovery of antibody reaction under the SWITCH-On condition. Our proposed single-step procedure thus serves as a generalized and practical protocol applicable to various dyes and antibodies.

Experimental conditions that can potentially affect the gel matrix structure, such as changes in gelatin concentration or limited digestion of extracellular matrix in tissue samples, have also been shown to influence the 3D immunostaining pattern[12] (Fig. 3b, e). We optionally incorporated a limited enzymatic reaction step into our current version of the protocol. In addition, temperature affected the efficiency of solute penetration[12] (Supplementary Fig. 3a). We set the appropriate temperature range from room temperature (approximately 25°C) to 37°C. Finally, the initial solute concentration was

essential (Supplementary Fig. 3a), as expected by the diffusion law[13] and our computational simulation (Supplementary Fig. 2a, b). Because the 3D immunostaining of the surrogate gelatin gel was saturated at an antibody concentration of more than 5 μg/mL (Supplementary Fig. 3a), we applied antibodies at concentrations in the range of 3.5 - 20 μg/mL for 3D immunostaining (Supplementary Data 1). This may also explain why some polyclonal antibodies, which contain multiple clones, were inferior to the monoclonal antibodies in terms of penetration efficiency or SBR in 3D staining (see Methods for details).

To better understand the basic principles of 3D staining, we modeled the procedure with the diffusion-reaction scheme as a first approximation (Supplementary Fig. 2a, b). Each experimental condition that we applied could solely or jointly modulate the terms R and D_{diff} in the formula. For example, the use of NaCl or Sca*l*eCUBIC-1A chemicals in the staining buffers could predominantly contribute to the term R by modulating the binding and unbinding kinetics of the stains. Since Quadrol and urea can cause swelling of the electrolyte gel due to their ability to keep solution alkaline (Fig. 1g) or interfere with hydrogen bonds[14, 15], respectively, these chemicals may also affect the D_{diff} term via the gel mesh structure η. However, because both dyes and antibodies can penetrate the gel even in the NaCl-induced shrinkage condition (Fig. 1g; Fig. 2b-e; Supplementary Fig. 3a), the physical mesh size of the gel does not seem to be problematic in our experiments. Indeed, the size of the mesh size of the delipidated brain was estimated to be approximately 30 nm according to SAXS analysis, which is similar to the width between the two Fab arms of the IgG molecule (on the order of 10 nm[16]).

Limited digestion of the ECM (Fig. 3e) could primarily regulate D_{diff} by changing the physical mesh structure η. The alteration of the gel structure might also modulate R by changing the strength of the physical and chemical interactions between the solute and the gel. The temperature could affect D_{diff} by altering the motion of the solute, as well as affecting the interactions represented in the R term. This might account for the apparent nonlinear change in the staining pattern between 4°C and 23°C (Supplementary Fig. 3a). Further comprehensive profiling will be required to precisely determine how these experimental conditions contribute to each theoretical parameter.

Preserved cellular-to-subcellular structures in the 3D-stained and cleared samples

Various features of the cellular-to-subcellular structures were preserved and captured by CUBIC-HV. For example, we successfully observed the distribution of somatostatin (Sst)-positive GABAergic cells in regions including cortical layers V/VI and the hypothalamus[17] (Supplementary Fig. 6a). We also detected ionized calcium binding

adapter molecule 1 (Iba1)-positive microglia distributed throughout the major regions of the brain[18] (Supplementary Fig. 6b, c). Large globular cells in the mesencephalic trigeminal nucleus, which were pan-neurofilament [Nf (pan)]- and parvalbumin (PV)-positive but phospho-neurofilament (phospho-Nf)-negative[19], could be successfully captured (Supplementary Fig. 6d-g). Furthermore, we identified several glial fibrillary acidic protein (GFAP)-positive cell foci in the cerebral cortex (Supplementary Fig. 6h). These foci may correspond to the assembly of gray matter astrocytes[20, 21] and potentially be related to the patchy astrogliosis observed in the brains of amyotrophic lateral sclerosis (ALS) or Alzheimer's disease patients[22, 23]. However, the physiological function of the observed foci remains to be elucidated. We observed afferent axons of tryptophan hydroxylase 2 (Tph2)-positive serotonergic neurons from the brainstem to the hypothalamic nuclei[24] (Supplementary Fig. 6i) and distributions of copeptin (a marker of vasopressin neuron)-positive neurites into and out of the hypothalamus, including a projection to the lateral habenula nucleus[25] (Supplementary Fig. 6j). Diverse distributions of MAP2-positive dendrites within the cortical areas (Supplementary Fig. 6k) and the dendritic arborizations of calbindin D28K-positive Purkinje cells (Supplementary Fig. 6l) were also captured. We further obtained LSFM and confocal microscope images of synaptophysin-stained samples, in which the expected synaptic staining patterns were visualized (Supplementary Fig. 6m, n). Therefore, organ-level 3D staining and imaging with CUBIC-HV can provide a wealth of information about the architecture of tissues and may lead to the discovery of hidden anatomical structures and cell populations.

Supplementary Methods

Step-by-step protocol of CUBIC-HistoVIsion (version 1.0) for a whole mouse brain

Note that this protocol will be updated. For inquiries, please contact the corresponding authors (suishess-kyu@umin.ac.jp or uedah-tky@umin.ac.jp).

Reagents

CUBIC-L

10 wt% *N*-Butyldiethanolamine (TCI #B0725)

10 wt% Triton X-100 (nacalai tesque #12967-45)

Dissolve in DDW

Sca*l*eCUBIC-1A with 500 mM NaCl

10 wt% Triton X-100 (nacalai tesque #12967-45)

5 wt% N,N,N',N'-Tetrakis(2-hydroxypropyl)ethylenediamine (Quadrol, TCI #T0781)

10 wt% Urea (nacalai tesque #35904-45)

500 mM NaCl (nacalai tesque #31319-45)

Dissolve in DDW

20 mg/mL Hyaluronidase stock (Sigma #H4272 or H3884)

Dissolve hyaluronidase in 50 mM Carbonate buffer, 150mM NaCl, 0.01% BSA* and 0.05% NaN_3** (pH 10).

*Sigma #A7906, add 1/100 volume of 1%(w/v) in water stock

**nacalai tesque #31208-82, add 1/200 volume of 10%(w/v) in water stock

Reaction buffer for Hyaluronidase (pH 10)*

10 mM CAPSO (Sigma #C2278)**

150 mM NaCl

0.05% NaN_3

*The pH is critical for giving a stringent reaction condition.

**0.5 M CAPSO buffer stock:

593.3 mg of CAPSO

DDW

Adjust pH to 10 with NaOH

==================================

Total 5 mL supplied with 0.05% NaN_3

Wash buffer for Hyaluronidase (pH 10)

50 mM Carbonate buffer*
150 mM NaCl*
0.1%(v/v) Triton X-100
5%(v/v) Methanol (nacalai tesque #21915-93)
0.05% NaN_3

*10x Carbonate buffer-NaCl stock:

2.96 g of Sodium Carbonate (nacalai tesque, #31310-35)
1.86 g of Sodium Hydrogen Carbonate (nacalai tesque, #31213-15)
8.77 g of NaCl (nacalai tesque, #31319-45)
DDW
Adjust pH to 10 with NaOH

==================================

Total 100 mL, supplied with 0.05% NaN_3

20 mg/mL Collagenase P stock (Sigma #11213857001)

Dissolve Collagenase P in HBSS (SIGMA, #H6648)

Reaction buffer for Collagenase P (pH 10)*

50 mM Carbonate buffer
150 mM NaCl
100 µM EDTA (nacalai tesque #15105-35)**
0.05% NaN_3
Dissolve in DDW

*The pH is critical for giving a stringent reaction condition.

**The concentration may be necessary for re-adjustment by every batch of the enzyme stock. We examined the range of 25-150 µM.

Wash buffer for Collagenase P (pH 10)

50 mM Carbonate buffer
150 mM NaCl
5 mM EDTA
5%(v/v) Methanol
0.05% NaN_3
Dissolve in DDW

Staining buffer (1x HEPES-TSC, pH 7.5)*

10 mM HEPES (nacalai tesque #17514-15)
10%(v/v) Triton X-100
200 mM NaCl
0.5%(w/v) Casein (Wako #030-01505)
0.05% NaN_3
Dissolve in DDW
* For preparing antibody solution, 2x HEPES-TSC is also recommended to be prepared. Occasionally, add Quadrol [2.5~5%, prepare by diluting 50 wt% in water stock] and/or urea (0.5~2 M, nacalai tesque #35904-45) for some antibodies.

0.5 M PB (pH 7.5) /500 mL

30.2 g Sodium hydrogen phosphate (nacalai tesque #31726-05)
5.9 g Sodium dihydrogenphosphate dehydrate (nacalai tesque #31718-15)
0.05% NaN_3
Dissolve in DDW

0.1 M PBT

0.1 M PB (pH 7.5)
10%(v/v) Triton X-100
0.05% NaN_3
Dissolve in DDW

1% Formaldehyde (FA)

0.1 M PB (pH 7.5)
1% Formaldehyde (nacalai tesque #16222-65, 37% saturated formaldehyde)
Dilute 1:37 formaldehyde in PB.

CUBIC-R+

45 wt% 2,3-Dimethyl-1-phenyl-5-pyrazolone (Antipyrine, TCI #D1876)
30 wt% Nicotinamide (TCI #N0078) or N-Methylnicotinamide (TCI #M0374)
0.5%(v/v) *N*-Butyldiethanolamine
Dissolve in DDW

Collection of mouse brains*

1. Anesthetize the mouse with an overdose of pentobarbital sodium salt (nacalai tesque #02095-04) in PBS or saline.
2. Transcardially perfuse with 10 mL of cold heparin-PBS.
3. Transcardially perfuse 20 mL of cold 4% (w/v) paraformaldehyde (PFA, nacalai tesque, #02890-45).
4. Dissect the brain from the skull.
5. Post-fix the dissected brain in 4% (w/v) PFA for overnight (8-24 h) at 4°C with gentle shaking.
6. Wash the sample in PBS for 3 h x 3 times at room temperature with gentle shaking.

*Refer to Susaki *et al*.[26] for details.

Delipidation with CUBIC-L

1*. Immerse a fixed whole mouse brain in 10 mL of 0.5x CUBIC-L (1:1 dilution with water) in a 30 mL tube (Sarstedt #60.544) for overnight at room temperature with gentle shaking.*
 *This step can be skipped.
2. Replace to 10-15 mL of 1x CUBIC-L in the 30 mL tube and delipidate for 3-5 days at 37°C with gentle shaking.
3. Wash the sample in PBS for 2 h x 3 times (or 2 h x1, overnight x1, 2 h x1) at 37°C with gentle shaking. The washing tube should be replaced with a new one every time to remove Triton X-100 intensively.

3D nuclear staining

1. Dilute either of nuclear stains in 4 mL of Sca*l*eCUBIC-1A with 500 mM NaCl.
 SYTOX™ Green (Thermo Fisher Scientific #S7020), 1:2500
 BOBO™-1 Iodide (462/481) (Thermo Fisher Scientific # B3582) 1:400
 RedDot™2 Far-Red Nuclear Stain (Biotium #40061), 1:150
2. Immerse the whole mouse brain in Sca*l*eCUBIC-1A with 500 mM NaCl containing either of the stains. Keep incubation with rotation at 37°C for the following periods: SYTOX-G, BOBO-1: 5 days, RedDot2: 3 days.
3. Wash the sample with 15 mL of 10 mM HEPES (pH 7.5) in a 30 mL tube for 2 h x 3 times at 25°C with gentle shaking.

Enzyme reaction (this step is considered as optional)

1. Immerse the whole mouse brain in 15 mL of reaction buffer in a 30 mL tube for overnight at 4°C with gentle shaking. (or for 2 h at 37°C)
2. Prepare hyaluronidase (final 3 mg/mL) or Collagenase P (final 1 mg/mL) in their reaction buffer.
3. Immerse the sample in the 500 μL* of enzyme solution in a 15 mL standing tube (Sarstedt #60.732.001) for 24 h at 37°C with gentle shaking.

   *The volumes and reaction time for smaller samples are as below:

Brain hemisphere	350 μL in 2 mL tube, the reaction for 18 h.
Cerebral hemispheres	250 μL in 2 mL tube, the reaction for 18 h.
Cerebellum hemispheres	120 μL in 2 mL tube, the reaction for 18 h.

4. Wash the sample in 15 mL of their wash buffer in a 30 mL tube for 2 h x 3 times at 37°C with gentle shaking.

3D immunostaining

1. To replace the buffer, immerse the whole mouse brain in 15 mL of the staining buffer (HEPES-TSC with additives, if needed) in a 30 mL tube for 1.5 h at a specific staining temperature for each antibody. Incubate it with gentle shaking.
2. Mix a primary antibody and a secondary Fab fragment in 50 μL of HEPES-TSC (without additive) in 0.5 mL tube (1:0.5 to 1:1 as the weight ratio). Incubate it for 1.5 h at 37°C with tapping and spinning down every 30 min.
3. Prepare 500 μL* of immunostaining solution by mixing the complex of primary antibody and secondary Fab, HEPES-TSC and additives if needed.

   A representative recipe is as below:

   Prepare the complex of primary antibody and secondary Fab fragment.

Primary antibody (1 mg/mL)	5 μL (= 5 μg)
Secondary Fab fragment (1.5 mg/mL)	3.3 μL (~ 5 μg for 1:1 weight ratio)
HEPES-TSC	50 μL
===	
	Total 58.3 μL

   Incubate it for 1.5 h at 37°C.

Prepare the immunostaining solution.

The antibody complex	58.3 µL
2x HEPES-TSC	225 µL
Additive	X µL**
Water	the remaining volume

==

Total 500 µL
(primary antibody: 5 µg in 500 µL = 10 µg/mL)

*The volumes for smaller samples are as below:

Brain hemisphere	350 µL
Cerebral hemispheres	250 µL
Cerebellum hemisphere	120 µL

**To supply the additive, use Quadrol [by diluting 50wt% stock to final 2.5-5%(v/v)] and urea (by diluting 5 M stock to final 0.5-2 M)

4. Immerse a whole mouse brain in the immunostaining solution in a 15 mL standing tube (Sarstedt #60.732.001). Keep the tube stood with gentle shaking (40-50 rpm) at a specific staining temperature for each antibody. The staining period is also dependent on each antibody (See Supplementary Table 1). To avoid the damage, put the brain so that the dorsal side comes to the bottom of the tube.
5. (optional) To improve the signal-noise ratio, incubate the sample in the immunostaining solution for 1 day~ at 4°C.
6. Wash the sample in 15 mL of 0.1 M PBT in the 30 mL tube for 30 min x 2 times at the incubated temperature with gentle shaking.
7. Wash the sample in 15 mL of 0.1 M PB in the 30 mL tube for 1 h at the same temperature with gentle shaking.

<u>Postfix with 1% FA</u>

1. Immerse the stained whole mouse brain in 1% FA in 0.1 M PB (8 mL in the 15 mL standing tube) for 24 h at 25°C with gentle shaking.
2. Wash the sample in 15 mL of 0.1M PB in a 30 mL tube for 2 h at 25°C with gentle shaking.

RI matching treatment and gel embedding

1. Immerse the whole mouse brain in 15 mL of 0.5x CUBIC-R+ (1:1 dilution with water) in a 30 mL tube for 24 h at 25°C with shaking.
2. Immerse a whole mouse brain in 30 mL of non-diluted CUBIC-R+ in 50 mL tube for 3 days at 25°C with gentle shaking.
3. After RI matching is completed, filtrate 15 mL of the used CUBIC-R+ with 5 µm filter (Millipore, #SLSVJ25LS). Store the brain sample in the remaining CUBIC-R+. Add 300 mg [2% (w/v)] of agar powder (nacalai tesque, #01163-76) into the filtrated CUBIC-R+. Completely disperse the agar powder in the reagent with a vortex.
4. Dissolve the ager by microwave. Repeat heating and mixing with vortex until the agar is completely dissolved.
6. Remove bubbles in 2% agar/CUBIC-R+ solution by incubation in a water bath for 5 min at 65°C.
7. For embedding the sample into a cleared gel, we use a Teflon mold (inside: W22 x D30 x H15 mm) of which the bottom is sealed with parafilm. Pre-heat the mold on a temperature-controlled plate (JUJI Field, LABOPAD C (COOL/HEAT), used with a custom-made black metal plate) at 45°C during step 6. Then, to make the bottom layer (approximately 2 mm thick), pour 2.5 mL of the agar solution into the pre-heated mold. Carefully remove bubbles at the surface of the gel by using a micropipette (the custom-made black metal plate and a strong light illumination help detect such small bubbles). The remaining gels should be kept in the water bath at 65°C.
8. Cool the plate for gelation for 15 min at 4°C. Then, warm the plate again for 5 min at 45°C.
9. Take out the RI-matched brain from the CUBIC-R+ and put it on a pre-heated (45°C) petri dish. Coat the brain with 1.5 mL of the agar solution.
10. Put the brain sample on the bottom layer of the gel so that the ventral side comes to the bottom. Pour the agar solution into the mold to almost cover the sample*. Remove bubbles by using a micropipette. The remaining gels should be kept in the water bath at 65°C.

    *An excess volume may cause the sample to float.
11. Cool the plate for gelation for 30 min at 4°C. Then, warm the plate again for 5 min at 45°C.
12. Pour a bit excess volume of the agar solution to make the top layer. Remove bubbles by using a micropipette. Then, seal the top layer of gel with a glass slide. Avoid making bubbles between the top layer of the gel and the glass slide.
13. Maintain the gel at 4°C for the complete gelation for about 1 h.

14. Unmold the sample-embedding gel at room temperature and immerse it in fresh CUBIC-R+ solution. Store it in the light-shielding container.

Supplementary References

1. Gradinaru, V., Treweek, J., Overton, K. & Deisseroth, K. Hydrogel-tissue chemistry: Principles and applications. *Annu. Rev. Biophys.* **47**, 355-376 (2018).
2. Hama, H. et al. ScaleS: an optical clearing palette for biological imaging. *Nat. Neurosci.* **18**, 1518-1529 (2015).
3. Murray, E. et al. Simple, Scalable Proteomic Imaging for High-Dimensional Profiling of Intact Systems. *Cell* **163**, 1500-1514 (2015).
4. Saito, T. et al. Single App knock-in mouse models of Alzheimer's disease. *Nat. Neurosci.* **17**, 661-663 (2014).
5. Annaka, M.T., T. Multiple phases of polymer gels. *Nature* **355**, 430-432 (1992).
6. Shibayama, M.I., F.; Inamoto, S.; Nomura, S. pH and salt concentration dependence of the microstructure of poly(N-isopropylacrylamide-co-acrylic acid) gels. *J. Chem. Phys.* **105**, 4358-4366 (1996).
7. Schaefer, D.W. Polymers, fractals, and ceramic materials. *Science* **243**, 1023-1027 (1989).
8. Beaucage, G. Small-Angle Scattering from Polymeric Mass Fractals of Arbitrary Mass-Fractal Dimension. *J. Appl. Cryst.* **29**, 134-146 (1996).
9. Susaki, E.A. et al. Whole-brain imaging with single-cell resolution using chemical cocktails and computational analysis. *Cell* **157**, 726-739 (2014).
10. Tainaka, K. et al. Whole-body imaging with single-cell resolution by tissue decolorization. *Cell* **159**, 911-924 (2014).
11. Tainaka, K. et al. Chemical Landscape for Tissue Clearing based on Hydrophilic Reagents. *Cell Rep.* **24**, 2196-2210 (2018).
12. Gleave, J.A., Lerch, J.P., Henkelman, R.M. & Nieman, B.J. A method for 3D immunostaining and optical imaging of the mouse brain demonstrated in neural progenitor cells. *PLoS ONE* **8**, e72039 (2013).
13. Cussler, E.L. Diffusion: Mass Transfer in Fluid Systems. (Cambridge University Press, 2009).
14. Hama, H. et al. Scale: a chemical approach for fluorescence imaging and reconstruction of transparent mouse brain. *Nat. Neurosci.* **14**, 1481-1488 (2011).
15. Murakami, T.C. et al. A three-dimensional single-cell-resolution whole-brain atlas using CUBIC-X expansion microscopy and tissue clearing. *Nat. Neurosci.* **21**, 625-637 (2018).
16. Murphy, R.M. et al. Size and structure of antigen-antibody complexes. Electron microscopy and light scattering studies. *Biophys. J.* **54**, 45-56 (1988).

17. Finley, J.C., Maderdrut, J.L., Roger, L.J. & Petrusz, P. The immunocytochemical localization of somatostatin-containing neurons in the rat central nervous system. *Neuroscience* **6**, 2173-2192 (1981).
18. Lawson, L.J., Perry, V.H., Dri, P. & Gordon, S. Heterogeneity in the distribution and morphology of microglia in the normal adult mouse brain. *Neuroscience* **39**, 151-170 (1990).
19. Ichikawa, H. et al. The number of nociceptors in the trigeminal ganglion but not proprioceptors in the mesencephalic trigeminal tract nucleus is reduced in dystonin deficient dystonia musculorum mice. *Brain Res.* **1226**, 33-38 (2008).
20. Houades, V. et al. Shapes of astrocyte networks in the juvenile brain. *Neuron Glia Biol.* **2**, 3-14 (2006).
21. Haas, B. et al. Activity-dependent ATP-waves in the mouse neocortex are independent from astrocytic calcium waves. *Cereb. Cortex* **16**, 237-246 (2006).
22. Kamo, H. et al. A distinctive distribution of reactive astroglia in the precentral cortex in amyotrophic lateral sclerosis. *Acta Neuropathol. (Berl)* **74**, 33-38 (1987).
23. Beach, T.G. & McGeer, E.G. Lamina-specific arrangement of astrocytic gliosis and senile plaques in Alzheimer's disease visual cortex. *Brain Res.* **463**, 357-361 (1988).
24. Azmitia, E.C. & Segal, M. An autoradiographic analysis of the differential ascending projections of the dorsal and median raphe nuclei in the rat. *J. Comp. Neurol.* **179**, 641-667 (1978).
25. Buijs, R.M. Intra- and extrahypothalamic vasopressin and oxytocin pathways in the rat. Pathways to the limbic system, medulla oblongata and spinal cord. *Cell Tissue Res.* **192**, 423-435 (1978).
26. Susaki, E.A. et al. Advanced CUBIC protocols for whole-brain and whole-body clearing and imaging. *Nat. Protoc.* **10**, 1709-1727 (2015).
