# Supplementary material for: Versatile whole-organ/body staining and imaging based on electrolyte-gel properties of biological tissues
Source: Nat Commun. 2020 Apr 27;11:1982. doi: 10.1038/s41467-020-15906-5 (PMC7184626; doi:10.1038/s41467-020-15906-5)
Supplement: Supplementary file 3 — Description of Additional Supplementary Files [file 41467_2020_15906_MOESM3_ESM.pdf]

Description of Additional Supplementary Files

File Name: Supplementary Data 1
Description: **Tested antibodies.** The tested antibodies and their evaluation results from 2D and 3D staining trials are summarized.

File Name: Supplementary Data 2
Description: **Summary of 3D staining and imaging conditions.** The organ-level 3D staining and imaging conditions shown in Fig. 3-9 are summarized.

File Name: Supplementary Movie 1
Description: **Whole-body 3D staining and imaging of infant marmoset, related to Fig. 2g-h.** The enlarged z-stack view of whole marmoset body staining and imaging in Fig. 2g-h. All the abdominal organs were uniformly stained and detected by the LSFM imaging. Note that the signal inside the vertebral canal was blurred because the bone had not been cleared by decalcification. The data was processed with Fiji/ImageJ.

File Name: Supplementary Movie 2
Description: **Representative whole-brain 3D staining and imaging (two colors), related to Fig. 4a**. Results of whole-brain staining and LSFM imaging with various antibodies (yellow) and nuclear stains (SYTOX-G or BOBO-1, blue) in Fig. 4a. The data were reconstituted and processed with Imaris software and Fiji/ImageJ.

File Name: Supplementary Movie 3
Description: **A fused, pseudo-multi-target whole-brain 3D staining, related to Fig. 4b.** Nine whole-brain imaging data in Fig. 4a (anti-phospho-Nf, calbindin D28K, PV, Sst, ChAT, Dbh, Th, Tph2 or copeptin antibodies with a nuclear stain) were registered and aligned to the whole-brain images of anti-NeuN antibody staining and nuclear staining (structural standard). The all transformed data were reconstituted and processed with Imaris software and Fiji/ImageJ.

File Name: Supplementary Movie 4
Description: **Multi-color and multi-modal whole-brain 3D staining and imaging, related to Fig. 5, 6 and Supplementary Fig. 8.** Quadruple co-staining images of the single whole adult mouse brain in Fig. 5e and Supplementary Fig. 8a [left: PV (Cy3), Sst (A594), Gad67 (A647) and BOBO-1, center: Sst (Cy3), ChAT (A594), Tph2 (A647) and BOBO-1] and a double antibody staining image of the single Thy1-YFP-H Tg whole adult mouse brain in Fig. 6a [right: ChAT (A594), Dat (A647) and YFP]. The data were reconstituted and processed with Imaris software and Fiji/ImageJ.

File Name: Supplementary Movie 5
Description: **Whole-hemisphere 3D staining and imaging of adult marmoset brain, related to Fig 9.** An adult marmoset brain hemisphere co-stained with SYTOX-G, GFAP (A594) and α-SMA (A647) antibodies in Fig. 9b. The data were reconstituted and processed with Imaris software and Fiji/ImageJ.
