# Supplementary material for: Versatile whole-organ/body staining and imaging based on electrolyte-gel properties of biological tissues
Source: Nat Commun. 2020 Apr 27;11:1982. doi: 10.1038/s41467-020-15906-5 (PMC7184626; doi:10.1038/s41467-020-15906-5)
Supplement: Supplementary file 11 — Reporting Summary [file 41467_2020_15906_MOESM11_ESM.pdf]

nature research

Corresponding author(s): Etsuo A. Susaki, Hiroki R. Ueda

Last updated by author(s): Jan 16, 2020

Reporting Summary

Nature Research wishes to improve the reproducibility of the work that we publish. This form provides structure for consistency and transparency in reporting. For further information on Nature Research policies, see Authors & Referees and the Editorial Policy Checklist.

Statistics

For all statistical analyses, confirm that the following items are present in the figure legend, table legend, main text, or Methods section.

n/a	Confirmed	
☐	☒	The exact sample size (n) for each experimental group/condition, given as a discrete number and unit of measurement
☐	☒	A statement on whether measurements were taken from distinct samples or whether the same sample was measured repeatedly
☐	☒	The statistical test(s) used AND whether they are one- or two-sided<br>*Only common tests should be described solely by name; describe more complex techniques in the Methods section.*
☒	☐	A description of all covariates tested
☐	☒	A description of any assumptions or corrections, such as tests of normality and adjustment for multiple comparisons
☐	☒	A full description of the statistical parameters including central tendency (e.g. means) or other basic estimates (e.g. regression coefficient) AND variation (e.g. standard deviation) or associated estimates of uncertainty (e.g. confidence intervals)
☐	☒	For null hypothesis testing, the test statistic (e.g. F, t, r) with confidence intervals, effect sizes, degrees of freedom and P value noted<br>*Give P values as exact values whenever suitable.*
☒	☐	For Bayesian analysis, information on the choice of priors and Markov chain Monte Carlo settings
☒	☐	For hierarchical and complex designs, identification of the appropriate level for tests and full reporting of outcomes
☒	☐	Estimates of effect sizes (e.g. Cohen's d, Pearson's r), indicating how they were calculated

Our web collection on statistics for biologists contains articles on many of the points above.

Software and code

Policy information about availability of computer code

Data collection	cellSens Dimension 1.18 (Olympus), Image Lab 4.1 (Bio-Rad), LabVIEW 2016 (National Instruments) for microscopy and gel imager operation, Mathematica 11 (Wolfram) for simulation
Data analysis	Igor Pro 6.3 (WaveMetrics)-based custom-written code for SAXS data analysis, Imaris 8.4 for 3D rendering of LSFM images, Fiji/ImageJ (latest versions when used) for reconstitution, preprocessing and cell detection of LSFM images (https://github.com/DSP-sleep/CUBIC-HistVIsion.git), ANTs (2.1.0.post690-g9e1c5) and MATLAB (R2015b) for registration and alignment of different brains (https://github.com/DSP-sleep/Landscape_pipeline/wiki), Excel for mac (Microsoft, latest versions when used) for data analysis and graph preparations, R (3.4.4 and 3.6.1) for statistical analyses and box plot preparation.

For manuscripts utilizing custom algorithms or software that are central to the research but not yet described in published literature, software must be made available to editors/reviewers. We strongly encourage code deposition in a community repository (e.g. GitHub). See the Nature Research guidelines for submitting code & software for further information.

Data

Policy information about availability of data

All manuscripts must include a data availability statement. This statement should provide the following information, where applicable:

- Accession codes, unique identifiers, or web links for publicly available datasets
- A list of figures that have associated raw data
- A description of any restrictions on data availability

The datasets used in the current study are available from the corresponding authors on reasonable request because the raw data files collected by light-sheet microscope are too large to upload (approximately two terabytes).

Field-specific reporting

Please select the one below that is the best fit for your research. If you are not sure, read the appropriate sections before making your selection.

☒ Life sciences ☐ Behavioural & social sciences ☐ Ecological, evolutionary & environmental sciences

For a reference copy of the document with all sections, see nature.com/documents/nr-reporting-summary-flat.pdf

Life sciences study design

All studies must disclose on these points even when the disclosure is negative.

Sample size	For measurement of delipidation procedure of the brains, n=3-4 was chosen as the replicate number. For measurement of swelling-shrinkage behaviors of the gels, n=3 was chosen as the replicate number. For measurement of EGFP signals (Tainaka et al. Cell Rep. 2018), n=3 was chosen as the replicate number and performed three independent experiments. We determined these sample sizes based on the literatures in the fields, and estimated that the variability of replicate was small enough to detect differences. No statistics were used to predetermine them.
Data exclusions	No data were excluded in the analyses.
Replication	The quantification of protein and lipid in the brain and the swelling-shrinkage assay of gels and tissues were repeated at least two independent samples. The SAXS analyses were performed with independent samples and detectors (SPring-8 and PF of KEK). Most of the staining and imaging experiments, except for marmoset hemisphere staining, were repeated with at least two independent samples in the same or comparable condition with slight modification (e.g., staining period). All the results were reliably reproduced. Since we obtained only one marmoset brain (two hemispheres) and the other one was used for determining the 3D staining condition with anti-alpha SMA antibody, we could not carry out another reproduction experiment for GFAP staining.
Randomization	The mice and an adult marmoset used for the experiments were randomly chosen from colonies. The infant marmoset was chosen due to neglect by the mother.
Blinding	No blinding was done in this study because knowledge of experimental conditions during data collection was required . Quantitative Analyses was conducted using semi-automated workflow.

Reporting for specific materials, systems and methods

We require information from authors about some types of materials, experimental systems and methods used in many studies. Here, indicate whether each material, system or method listed is relevant to your study. If you are not sure if a list item applies to your research, read the appropriate section before selecting a response.

Materials & experimental systems

n/a	Involved in the study	
☐	☒	Antibodies
☒	☐	Eukaryotic cell lines
☒	☐	Palaeontology
☐	☒	Animals and other organisms
☐	☒	Human research participants
☒	☐	Clinical data

Methods

n/a	Involved in the study	
☒	☐	ChIP-seq
☒	☐	Flow cytometry
☒	☐	MRI-based neuroimaging

Antibodies

Antibodies used

anti-NeuN, Millipore, MAB377 (Lot# 2592741)
anti-NeuN, Millipore, ABN78 (Lot# 2885346)
anti-Calbindin D28K, Sigma, C2724 (Lot# 093M4801)
anti-Parvalbumin (PV), Swant, PV235 (Lot# 10-11(F))
anti-Somatostatin (Sst), Peninsula, T4103 (Lot# A14111)
anti-Somatostatin (Sst), Millipore, MAB354, clone YC7 (Lot# 3005269)
anti-Glutamic Acid Decarboxylase (Gad) 67 specific, Millipore, MAB5406, clone 1G10.2 (Lot# 2844575)
anti-Glutamic Acid Decarboxylase (Gad) 65/67, Sigma, G5163
anti-Glutamic Acid Decarboxylase (Gad) 65/67, MBL, M018-3 (Lot# 024)
anti-Tyrosine hydroxylase (Th), Santa Cruz, sc-25269 (Lot# H2510)
anti-Dopamine Transporter (Dat), Sigma, HPA013602 (Lot# A97032)
anti-Dopamine Transporter (Dat), abcam, ab128848 (Lot# GR249640-3)
anti-Dopamine beta Hydroxylase (Dbh), abcam, ab209487 (Lot# GR310529-2)
anti-Choline Acetyltransferase (ChAT), Millipore, AB144 (Lot# 3018862)

anti-Choline Acetyltransferase (ChAT), abcam, ab178850 (Lot# GR315911-3)
anti-Tryptophan hydroxylase 2 (Tph2), Sigma, AMAb91108 (Lot# 02981)
anti-Copeptin, Santa Cruz, sc-7812 (Lot# J0604)
anti-LRPAP-1, Sigma, HPA008001 (Lot# R02480)
anti-Camk2b, Sigma, HPA026307 (Lot# R27007)
anti-RAP1GAP, Sigma, HPA001922 (Lot# R03996)
anti-p75NTR, MBL, D297-3 (Lot# 001)
anti-PKCa/PRKCA1, LSBio, LS-C164546 (Lot# 75278)
anti-PKCa/PRKCA1, Novus, NB600-201 (Lot# A-2)
anti-Ionized calcium binding adapter molecule 1 (Iba1), Wako, 019-19741 (Lot# CTF4377)
anti-Glial Fibrillary Acidic Protein (GFAP), Sigma, C9205 (Cy3 conj) (Lot# 022M4752V)
anti-Glial Fibrillary Acidic Protein (GFAP), MBL, D097-3 (Lot# 012)
anti-Oligodendrocyte transcription factor 2 (Olig2), IBL, 18953 (Lot# 1D-104)
anti-Oligodendrocyte transcription factor 2 (Olig2), Abcam, ab109186 (Lot# GR210294-11)
anti-Neurofilament (pan), invitrogen, 18-0171Z (Lot# 1634056A)
anti-Neurofilament (L+H), Monosan, MON 3004 (Lot# 373004E)
anti-Phospho-Neurofilament, Biolegend, 801601, SMI31 (Lot# B222936)
anti-Microtubule-Associated Protein (MAP) 2, Sigma, HPA012828 (Lot# A96874)
anti-Microtubule-Associated Protein (MAP) 2, Abcam, ab11267 (Lot# GR319463-3)
anti-Synaptophysin, MBL, D073-3 (Lot# 013)
anti-Synaptotagmin1/2, MBL, D156-3 (Lot# 011)
anti-Synapsin-1, Millipore, AB1543P (Lot# 2195992)
anti-α-Smooth Muscle Actin (SMA), Sigma, A5228 (Lot# 074M4814V)
anti-c-Fos, Sigma, HPA018531 (Lot# E105612)
anti-c-Fos, Abcam, clone 2H2 / ab208942(Lot# GR3187875-2)
anti-c-Fos, CST, 2250S (Lot# 9)
anti-Arc, Synaptic Systems, 156 003 (Lot# 156003/1-63)
anti-β-Amyloid (6E10), Biolegend, 93049 (Lot# B213174)
anti-β-Amyloid (82E1), IBL, 10323 (Lot# 0D-906)
anti-NeuN (A488-conjugated, Merck Millipore, MAB377X)
anti-CREB, CST, #9197
anti-mouse secondary IgG (Thermo Fisher Scientific, #A11029 for Alexa 488, #A21203 for Alexa 594)
anti-rabbit secondary IgG (Thermo Fisher Scientific, #A21206 for Alexa 488, #A10040 for Alexa 546, #A21207 for Alexa 594, #A31573 for Alexa 647)
FabuLightTM AffiniPure Fab Fragment, Fc fragment specific [Jackson ImmunoResearch laboratories, anti-Mouse IgG1 (#115-547-185 for Alexa 488, #115-167-185 for Cy3, #115-587-185 for Alexa 594, #115-607-185 for Alexa 647), anti-Mouse IgG2a (#115-587-186 for Alexa 594, #115-607-186 for Alexa 647), anti-Mouse IgG2b (#115-587-187 for Alexa 594), Anti-Rat IgG1 (#112-167-008 for Cy3, #112-587-008 for Alexa 594), Anti-Rabbit IgG (#111-167-008 for Cy3, #111-587-008 for Alexa 594, #111-607-008 for Alexa 647), and Anti-Goat IgG (#805-587-008 for Alexa 594)]

Validation

Antibody validation has been done according to the vender's information on reacting species and applications and by staining a thin-slice section with CUBIC-HV staining buffers (Supplementary Fig. 5), which were occasionally comparing the staining pattern to established scientific papers and vender's catalog that performed immunostaining for the same targets. Subsequently, 3D whole-brain antibody staining was performed, and the staining was similarly inspected and validated.
The list of vender's website for each antibody are as below:
• anti-NeuN, Millipore, MAB377 and MAB377X, reactivity: including mouse, application: including ICC/IF/IHC
https://www.emdmillipore.com/US/en/product/NeuN-Antibodies,MM_NF-C87804
• anti-NeuN, Millipore, ABN78, reactivity: including mouse, application: including ICC/IHC
https://www.merckmillipore.com/INTL/en/product/Anti-NeuN-Antibody-rabbit,MM_NF-ABN78
• anti-Calbindin D28K, Sigma, C2724, reactivity: including rat (a conserved c-terminal sequence was used for immunogen), application: including IHC
https://www.sigmaaldrich.com/catalog/product/sigma/c2724?lang=en&region=CA
• anti-Parvalbumin (PV), Swant, PV235, reactivity: including mouse, application: including IHC (validated by KO mouse)
https://www.swant.com/pdfs/Monoclonal_parvalbumin_235.pdf
• anti-Somatostatin (Sst), Peninsula, T4103, reactivity: no documentation (validated by the authors), application: including IHC
http://www.bma.ch/files/product/t-4103.pdf
• anti-Somatostatin (Sst), Millipore, MAB354, clone YC7, reactivity: including rat (used on mice in Nature (2015) 520, 499–504 and other publications), application: including IHC
https://www.merckmillipore.com/INTL/en/product/Anti-Somatostatin-Antibody-clone-YC7,MM_NF-MAB354
• anti-Glutamic Acid Decarboxylase (Gad) 67 specific, Millipore, MAB5406, clone 1G10.2, reactivity: including mouse, application: including IHC
https://www.merckmillipore.com/INTL/en/product/Anti-GAD67-Antibody-clone-1G10.2,MM_NF-MAB5406
• anti-Glutamic Acid Decarboxylase (Gad) 65/67, Sigma, G5163, reactivity: including mouse, application: including IHC
https://www.sigmaaldrich.com/catalog/product/sigma/g5163?lang=en&region=CA
• anti-Glutamic Acid Decarboxylase (Gad) 65/67, MBL, M018-3, reactivity: including mouse, application: including IHC
https://www.mblintl.com/products/m018-3
• anti-Tyrosine hydroxylase (Th), Santa Cruz, sc-25269, reactivity: including mouse, application: including IF/IHC
https://datasheets.scbt.com/sc-25269.pdf
• anti-Dopamine Transporter (Dat), Sigma, HPA013602, reactivity: including human*, application: including IHC
https://www.sigmaaldrich.com/catalog/product/sigma/hpa013602?lang=en&region=CA
*Comparable brain areas and cells labeled by anti-Dat (abcam, ab128848) were stained.
• anti-Dopamine Transporter (Dat), abcam, ab128848, reactivity: including mouse, application: including IHC
https://www.abcam.com/dopamine-transporter-antibody-6v-23-23-ab128848.html
• anti-Dopamine beta Hydroxylase (Dbh), abcam, ab209487, reactivity: including mouse, application: including IHC

https://www.abcam.com/dopamine-beta-hydroxylase-antibody-epr20385-ab209487.html
• anti-Choline Acetyltransferase (ChAT), Millipore, AB144P, reactivity: including mouse, application: including IHC
https://www.merckmillipore.com/JP/en/product/Anti-Choline-Acetyltransferase-Antibody,MM_NF-AB144P
• anti-Choline Acetyltransferase (ChAT), abcam, ab178850, reactivity: including mouse, application: including IHC
https://www.abcam.co.jp/choline-acetyltransferase-antibody-epr16590-ab178850.html ※Japan page
• anti-Tryptophan hydroxylase 2 (Tph2), Sigma, AMAb91108, reactivity: including mouse, application: including IHC
https://www.sigmaaldrich.com/catalog/product/sigma/amab91108?lang=en&region=CA
• anti-Copeptin, Santa Cruz, sc-7812, reactivity: including mouse, application: including IF
https://datasheets.scbt.com/sc-7812.pdf
• anti-LRPAP-1, Sigma, HPA008001, reactivity: including mouse, application: including IHC
https://www.sigmaaldrich.com/catalog/product/sigma/hpa008001?lang=en&region=CA
• anti-Camk2b, Sigma, HPA026307, reactivity: including mouse, application: including IHC
https://www.sigmaaldrich.com/catalog/product/sigma/hpa026307?lang=en&region=US
• anti-RAP1GAP, Sigma, HPA001922, reactivity: including mouse, application: including IHC
https://www.sigmaaldrich.com/catalog/product/sigma/hpa001922?lang=en&region=US
• anti-p75NTR, MBL, D297-3, reactivity: including mouse, application: flow cytometry**
https://www.mblintl.com/products/d297-3
**no specific staining on the mouse brain sample
• anti-PKCa/PRKCA1, LSBio, LS-C164546, reactivity: including mouse, application: including IHC
(discontinued product)
• anti-PKCa/PRKCA1, Novus, NB600-201, reactivity: including mouse, application: including ICC/IF/IHC
https://www.novusbio.com/products/pkc-alpha-antibody-mc5_nb600-201
• anti-Ionized calcium binding adapter molecule 1 (Iba1), Wako, 019-19741, reactivity: including mouse, application: including ICC/IHC
https://labchem-wako.fujifilm.com/us/category/01213.html
• anti-Glial Fibrillary Acidic Protein (GFAP), Sigma, C9205 (Cy3 conj), reactivity: including human and rat**, application: including IF
https://www.sigmaaldrich.com/catalog/product/sigma/c9205?lang=en&region=CA
• anti-Glial Fibrillary Acidic Protein (GFAP), MBL, D097-3, reactivity: human**, application: including ICC
https://www.mblintl.com/products/d097-3
**The staining patterns were comparable to previous reports, such as PLoS ONE (2013) 8, e72039.
• anti-Oligodendrocyte transcription factor 2 (Olig2), IBL, 18953, reactivity: including mouse, application: including IHC
https://www.ibl-japan.co.jp/en/search/product/detail/id=3776
• anti-Oligodendrocyte transcription factor 2 (Olig2), Abcam, ab109186, reactivity: including mouse, application: including IHC
https://www.abcam.com/olig2-antibody-epr2673-ab109186.html
• anti-Neurofilament (pan), invitrogen, 18-0171Z, reactivity: including rat***, application: including IHC
https://www.labome.com/product/Invitrogen/180171Z.html
***Axons and specific neurofilament-positive cells [e.g., neurons in trigeminal nucleus, BBRC (1988) 154, 1099-1106] were observed on mouse brain slices.
• anti-Neurofilament (L+H), Monosan, MON 3004, reactivity: including mouse, application: including IHC
https://www.monosan.com/mon3004-1
• anti-Phospho-Neurofilament, Biolegend, 801601, SMI31, reactivity: including mouse, application: including IHC
https://www.biolegend.com/en-us/products/purified-anti-neurofilament-h-nf-h-phosphorylated-antibody-11476
• anti-Microtubule-Associated Protein (MAP) 2, Sigma, HPA012828, reactivity: human****, application: including IF/IHC
https://www.sigmaaldrich.com/catalog/product/sigma/hpa012828?lang=en&region=CA
****Comparable brain areas and structures labeled by anti-MAP2 (abcam, ab11267) were stained.
• anti-Microtubule-Associated Protein (MAP) 2, Abcam, ab11267, reactivity: including mouse, application: including ICC/IF/IHC
https://www.abcam.com/map2-antibody-hm-2-ab11267.html
• anti-Synaptophysin, MBL, D073-3, reactivity: human‡, application: including IHC
https://www.mblintl.com/products/d073-3
‡The authors confirmed the synaptic staining pattern on mouse brain samples.
• anti-Synaptotagmin1/2, MBL, D156-3, reactivity: rat, application: including IC
(Discontinued product)
• anti-Synapsin-1, Millipore, AB1543P, reactivity: including mouse, application: including ICC/IHC
https://www.merckmillipore.com/INTL/en/product/Anti-Synapsin-I-Antibody,MM_NF-AB1543P
• anti-α-Smooth Muscle Actin (SMA), Sigma, A5228, reactivity: including mouse, application: including ICC/IF/IHC
https://www.sigmaaldrich.com/catalog/product/sigma/a5228?lang=en&region=CA
• anti-c-Fos, Sigma, HPA018531, reactivity: human‡‡, application: including IF/IHC
https://www.sigmaaldrich.com/catalog/product/sigma/hpa018531?lang=en&region=CA
‡‡The authors confirmed positive nuclei staining in MK-801-stimulated mouse brain sections.
• anti-c-Fos, Abcam, clone 2H2 / ab208942, reactivity: including mouse, application: including ICC/IF/IHC
https://www.abcam.com/c-fos-antibody-2h2-ab208942.html
• anti-c-Fos, CST, 2250S, reactivity: including mouse, application: including IF
https://www.cellsignal.jp/products/primary-antibodies/c-fos-9f6-rabbit-mab/2250
• anti-Arc, Synaptic Systems, 156 003, reactivity: including mouse, application: including ICC/IHC
https://www.sysy.com/products/arc/facts-156003.php
• anti-β-Amyloid (6E10), Biolegend, 93049, reactivity: human [used in the AD mouse model (Nat Neurosci. (2014) 17,661–663], application: including ICC/IHC
https://www.biolegend.com/en-us/products/purified-anti-beta-amyloid-1-16-antibody-11228
• anti-β-Amyloid (82E1), IBL, 10323, reactivity: human [used in the AD mouse model (Nat Neurosci. (2014) 17,661–663], application: including IP/IHC
https://www.ibl-japan.co.jp/en/search/product/detail/id=3554

- anti-CREB, CST, #9197, reactivity: including mouse, application: including ICC/IHC https://www.cellsignal.jp/products/primary-antibodies/creb-48h2-rabbit-mab/9197

Animals and other organisms

Policy information about studies involving animals; ARRIVE guidelines recommended for reporting animal research

Laboratory animals	8-week-old male C57BL/6N mice<br>8-month-old female Thy1-YFP-H transgenic mouse<br>9-month-old male App(NL-G-F) knock-in mouse (RBRC06344, RIKEN BRC)<br>8-week-old male Gad2-Cre knock-in mouse [Gad2(tm2(cre)Zjh)/J, Jackson laboratory #010802]<br>Marmosets, an infant (postnatal day 1, gender undetermined) and adults (6-year-old male and 7-years-old female)
Wild animals	No wild animal was used.
Field-collected samples	No field-collected samples were used.
Ethics oversight	Animal Care and Use Committees of Graduate School of Medicine and Graduate school of Agricultural and Life Sciences of the University of Tokyo, RIKEN Kobe institute, National Institute for Physiological Sciences, and Graduate School of Medicine of Kyoto University.

Note that full information on the approval of the study protocol must also be provided in the manuscript.

Human research participants

Policy information about studies involving human research participants

Population characteristics	A male Japanese patient who underwent pathological dissection at Niigata University Hospital, who had suffered amyotrophic lateral sclerosis and died of respiratory failure at the age of 73 years.
Recruitment	The autopsies were performed at the Brain Research Institute, Niigata University for evaluating the precise clinicopathological features of deceased individuals. Written informed consent for autopsy including the use of tissue for research purposes was obtained from the next of kin. For histopathological evaluation, we routinely immerse autopsied brain in formalin for four weeks. Then, we made several thick sagittal slices of the cerebellum taken from the autopsied patient, and then we processed the slices for tissue clearing. Therefore, there is no case of selection bias. The patient's brain was chosen because of no detectable histopathological alteration in his cerebellum. The fixation time may affects clearing efficiency and penetration of the dye and the antibodies.
Ethics oversight	Ethical Review Boards of Brain Research Institute, Niigata University (No. 1992) and Graduate School of Medicine of The University of Tokyo (No. 10544 and No.10714)

Note that full information on the approval of the study protocol must also be provided in the manuscript.
